# Graphical calibration curves and the integrated calibration index (ICI) for competing risk models

**DOI:** 10.1186/s41512-021-00114-6

**Published:** 2022-01-17

**Authors:** Peter C. Austin, Hein Putter, Daniele Giardiello, David van Klaveren

**Affiliations:** 1 ICES, G106, 2075 Bayview Avenue, Toronto, Ontario M4N 3M5 Canada; 2 Institute of Health Management, Policy and Evaluation, University of Toronto, Toronto, Ontario Canada; 3 Sunnybrook Research Institute, Toronto, Ontario Canada; 4 Department of Biomedical Data Sciences, Leiden University Medical Centre, Leiden, The Netherlands; 5 Division of Molecular Pathology, The Netherlands Cancer Institute - Antoni van Leeuwenhoek Hospital, Amsterdam, The Netherlands; 6 Institute for Biomedicine (affiliated with the University of Lübeck), Eurac Research, Bolzano, Italy; 7 Department of Public Health, Erasmus MC, Rotterdam, The Netherlands; 8 Predictive Analytics and Comparative Effectiveness Center, Institute for Clinical Research and Health Policy Studies, Tufts Medical Center, Boston, USA

**Keywords:** Calibration, Competing risks, Survival analysis, Time-to-event model, Model validation, Random forests

## Abstract

**Background:**

Assessing calibration—the agreement between estimated risk and observed proportions—is an important component of deriving and validating clinical prediction models. Methods for assessing the calibration of prognostic models for use with competing risk data have received little attention.

**Methods:**

We propose a method for graphically assessing the calibration of competing risk regression models. Our proposed method can be used to assess the calibration of any model for estimating incidence in the presence of competing risk (e.g., a Fine-Gray subdistribution hazard model; a combination of cause-specific hazard functions; or a random survival forest). Our method is based on using the Fine-Gray subdistribution hazard model to regress the cumulative incidence function of the cause-specific outcome of interest on the predicted outcome risk of the model whose calibration we want to assess. We provide modifications of the integrated calibration index (ICI), of E50 and of E90, which are numerical calibration metrics, for use with competing risk data. We conducted a series of Monte Carlo simulations to evaluate the performance of these calibration measures when the underlying model has been correctly specified and when the model was mis-specified and when the incidence of the cause-specific outcome differed between the derivation and validation samples. We illustrated the usefulness of calibration curves and the numerical calibration metrics by comparing the calibration of a Fine-Gray subdistribution hazards regression model with that of random survival forests for predicting cardiovascular mortality in patients hospitalized with heart failure.

**Results:**

The simulations indicated that the method for constructing graphical calibration curves and the associated calibration metrics performed as desired. We also demonstrated that the numerical calibration metrics can be used as optimization criteria when tuning machine learning methods for competing risk outcomes.

**Conclusions:**

The calibration curves and numeric calibration metrics permit a comprehensive comparison of the calibration of different competing risk models.

**Supplementary Information:**

The online version contains supplementary material available at 10.1186/s41512-021-00114-6.

## Background

Assessing calibration is an important component of deriving and validating clinical prediction models. Calibration refers to the agreement between predicted and observed risk [1, 2]. Methods for assessing calibration for models for binary outcomes and for models for time-to-event outcomes in the absence of competing risks have been described previously [1–5].

In the analysis of time-to-event outcomes, a competing risk is an event whose occurrence precludes the occurrence of the event of primary interest [6]. For example, in a study in which the primary outcome is cardiovascular death, non-cardiovascular death is a competing risk since those who die of non-cardiovascular causes are no longer at risk of death due to cardiovascular causes. There is little information on methods to assess the calibration of competing risk regression models (i.e., models for time-to-event outcomes in the presence of competing risks) [7, 8].

When outcomes are time-to-event in nature, the objective of prognostic models is frequently focused on estimating the probability of the occurrence of the outcome within a specified duration of time (i.e., the cumulative incidence function (CIF)). In the presence of competing risks, the complement of the Kaplan-Meier estimate of the survival function results in biased estimation of the CIF. Similarly, using a single Cox proportional hazards model or a single cause-specific hazard model will result in biased estimation of the CIF [6, 9, 10]. Instead, the CIF should be used to estimate crude incidence. In multivariable settings, one can use a Fine-Gray subdistribution hazard model to estimate the incidence of time-to-event outcomes over time in the presence of competing risks [6, 9, 10]. The principle alternative to using a Fine-Gray subdistribution hazard model is to fit separate cause-specific hazard models for each of the types of outcomes and calculate the CIF by combining the individual cause-specific hazard models [10]. Since conventional methods for the analysis of survival data cannot be used for predicting incidence in the presence of competing risks, methods for assessing calibration in the absence of competing risks are not applicable to settings with competing risks.

Methods for assessing the calibration of prognostic models for use with competing risk data have received little attention. To the best of our knowledge, we are aware of only two described approaches for assessing calibration of competing risk methods. First, Wolbers and colleagues categorized subjects according to percentiles of predicted risk [7]. Within each risk stratum, they computed the mean predicted risk and the empirically observed risk (estimated using a CIF) and then plotted observed risk versus mean predicted risk across the risk strata. Second, Gerds and colleagues described a method for creating calibration curves for competing risks models based on jackknife pseudo-values combined with a nearest neighbour smoother [8]. While the former approach requires categorization of risk through the creation of risk strata, the latter approach uses a smoother based on the observed risk for each subject.

The objective of this paper is to describe and evaluate a new method for graphically and numerically assessing the calibration of competing risk models. The paper is structured as follows: In “Methods for assessing calibration of models for binary and time-to-event outcomes in the absence of competing risks”, we summarize common methods for assessing calibration of models for binary outcomes and models for time-to-event outcomes in the absence of competing risks. In “Graphical calibration curves and calibration metrics for competing risk models”, we describe a method to compute smoothed calibration curves for competing risk regression models. We also describe how to compute numeric metrics for summarizing calibration of competing risk regression models. In “Monte Carlo simulations: Methods”, we describe a series of Monte Carlo simulations to evaluate the ability of this method to assess calibration. In “Monte Carlo simulations: Results”, we report the results of these simulations. In “Case study”, we present a case study illustrating the application of these methods when comparing the calibration of two subdistribution hazard models for predicting the incidence of cardiovascular mortality after hospitalization for heart failure. Finally, in “Conclusion”, we summarize our findings and place them in the context of the literature.

## Methods for assessing calibration of models for binary and time-to-event outcomes in the absence of competing risks

### Calibration for binary events

When outcomes are binary, calibration refers to the agreement between observed proportions and estimated probabilities of the occurrence of the event or outcome. Several methods have been proposed to assess calibration in this setting. First, the calibration intercept and slope can be used [1, 2, 4]. These are estimated by using logistic regression to regress the binary outcome on the estimated linear predictor. These indicate whether the mean predicted probability accurately estimates the overall empirical probability of the outcome and whether predicted probabilities display too little or too much variation. Second, subjects can be divided into strata based on the predicted probability of the outcome (e.g., dividing subjects into ten equally sized groups using the deciles of the predicted probabilities). Then, within each stratum, the mean predicted probability is computed along with the empirically estimated probability of the outcome (i.e., the crude estimated probability of the outcome amongst all subjects in the given stratum; this is the observed probability of the outcome). The mean predicted probability of the outcome (“predicted risk”) can then be compared with the observed probability of the outcome (“observed risk”) across strata. These can be compared graphically, with deviations from a diagonal line denoting lack of calibration. While this approach is simple to implement, a limitation is the potential loss of information resulting from binning subjects into strata based on predicted risk. However, the comparison of predicted and observed risk serves as a motivation for the third method. Third, rather than dividing subjects into strata based on the predicted probability of the outcome, smooth calibration curves based on local polynomial regression (e.g., locally estimated scatterplot smoothing (loess)) or flexible nonlinear models can be produced [1–3]. This approach does not require dividing subjects into risk strata but is based on estimating “observed” risk using the loess regression smoother (or a flexible nonlinear model). Thus, the “observed” risk is the estimated probability of the outcome for a given value of predicted risk, based on the loess regression smoother (this model-based estimate of “observed” risk will serve as a motivation for the adaption of this approach for use with competing risks). This allows for an assessment of the agreement between observed and predicted risk across the spectrum of predicted risk, rather than across a limited number of risk categories. Fourth, numeric summary measures of calibration, such as the integrated calibration index (ICI), E50, E90, and *E*_max_ can be reported [1, 11]. The ICI is the weighted difference between smoothed observed proportions and predicted probabilities, in which observations are weighted by the empirical density function of the predicted probabilities. The ICI is equivalent to the mean difference between predicted probabilities and observed probabilities derived from a smoothed calibration curve. E50 and E90 denote the median and 90th percentile of the absolute difference between observed and predicted probabilities. *E*_max_ denotes the maximum absolute difference between observed and predicted probabilities of the outcome.

### Extensions to survival outcomes in the absence of competing risks

When outcomes are time-to-event in nature, calibration typically refers to the agreement between observed and estimated probabilities of the occurrence of the event within a specified duration of time. Thus, if multiple time points are of interest clinically, one would need to assess calibration at each of these time points. The most commonly used approach for assessing calibration appears to be a modification of the stratification-based approach described above for use with binary outcomes [1]. Subjects are divided into strata based on the predicted probability of the occurrence of the event by time *t*. Within each stratum, the mean predicted probability of the occurrence of the event by time *t* is computed. Then, within each stratum, the observed probability of the occurrence of the event by time *t* is computed using the complement of the Kaplan-Meier survival function fit to the subjects in that stratum. The mean predicted and observed probabilities can then be compared across strata, possibly using a scatter plot and superimposing a diagonal line on the resultant plot. A limitation of this approach is that, in addition to the risk categories being arbitrary, the categorization of predicted risk can lead to a loss of precision [1] (page 506). Harrell, Crowson and colleagues, and Austin and colleagues have described methods to create smoothed calibration curves for time-to-event models [1, 5, 12]. These curves are modifications of the smoothed calibration curves for binary outcomes that have been adapted for use with time-to-event outcomes. The method recently described by Austin and colleagues was motivated by the use of locally estimated scatterplot smoothing (loess) for assessing the calibration of models for binary outcomes. The hazard of the outcome is regressed on the predicted outcome risk using a flexible regression model. As with the loess-based approach for binary models, the “observed” outcome risk is the model-based estimate of outcome risk for the given value of predicted outcome risk.

## Graphical calibration curves and calibration metrics for competing risk models

In this section, we describe methods for constructing smoothed calibration plots for competing risk models and how numerical calibration metrics can be derived from these smoothed calibration curves. The described approach is motivated by the recently proposed methods for creating smoothed calibration curves for time-to-event models in the absence of competing risks [5].

### Graphical calibration curves

Motivated by comparable methods for binary outcomes and for survival outcomes in the absence of competing risks, our objective is to compare the agreement between predicted and observed risk across the range of predicted risk. Model-based estimates will be obtained of the observed risk of the outcome of interest in the presence of competing risks.

Let *F*_1_(*t*_0_| **X**) denote a model for estimating the cause-specific cumulative incidence of the occurrence of an event prior to time *t*_0_ for a subject with covariate vector **X** (we use the subscript “1” to denote that we are modelling the cause-specific cumulative incidence of type 1 events, accounting for competing risks of other event types). While* F*_1_(*t*_0_| **X**), the estimate of predicted risk, would often be estimated using a Fine-Gray subdistribution hazard model [13], other methods, including fitting the cause-specific hazard models for all the different types of events and combining the cause-specific hazard functions can also be used [10]. We highlight the Fine-Gray subdistribution hazard model as it is the most frequently used method for estimating incidence in the presence of competing risk. For each subject, let ${\hat{\mathrm{I}}}_{t_0}={F}_1\left({t}_0|\mathbf{X}\right)$ denote the predicted probability of the occurrence of the outcome prior to time *t*_0_ (i.e., the estimated cumulative incidence function).

Our graphical method for assessing calibration involves comparing the agreement between predicted and observed risk. Our approach for estimating observed risk is motivated by the loess-based approach described above for binary outcomes and by the modification of that method that we have described for use with survival outcomes in the absence of competing risks. In both of those settings, a regression model was used to regress the outcome (or the hazard of the outcome) on predicted risk (i.e., the predicted risk obtained from the model whose calibration we want to assess). The resultant model-based estimate was used as the value of observed risk corresponding to the given value of predicted risk. Once the model whose calibration we want to assess has been fit (note the prediction model whose calibration we want to assess can be any type of model that allows for estimating cumulative incidence in the presence of competing risks, not necessarily a Fine-Gray subdistribution hazard model), we propose using a Fine-Gray subdistribution hazards model with restricted cubic splines to model the relationship between the logarithm of the subdistribution hazard of the cause-specific outcome and the complementary log-log transformation of the predicted probability of the cause-specific outcome occurring prior to time *t*_0,_ that is $\log \left(-\log \left(1-{\hat{\mathrm{I}}}_{t_0}\right)\right)$ (i.e., the sole independent variable is the complementary log-log transformation of the predicted probability of the cause-specific outcome that was obtained from the model whose calibration we want to assess). Based on the fitted model, the probability of the occurrence of the cause-specific outcome prior to time *t*_0_ can be estimated for each value of ${\hat{\mathrm{I}}}_{t_0}$(note that the predicted probability ${\hat{\mathrm{I}}}_{t_0}$was obtained using the model fit in the previous paragraph, whose calibration one wants to assess). Analogous with what was done for binary outcomes and survival outcomes in the absence of competing risks, these estimates serve as the estimates of observed risk. From these estimated probabilities, a calibration curve can be constructed comparing predicted and observed risk. Note that we use ${\hat{\mathrm{I}}}_{t_0}$in the preceding function, to highlight that calibration is being assessed at time *t*_0_, with ${\hat{\mathrm{I}}}_{t_0}$denoting the predicted probability of a cause-specific event occurring prior to time *t*_0_. Furthermore, while the model regressed the subdistribution hazard of the cause-specific outcome on the complementary log-log transformation of the predicted probability, we report results on the probability scale for greater interpretability. For each observed value of ${\hat{\mathrm{I}}}_{t_0}$, the estimated probability of the occurrence of the cause-specific outcome occurring prior to time *t*_0_ is obtained. These are displayed graphically to produce a calibration plot for time *t*_0_.

### Numerical metrics for calibration

Once a smoothed calibration curve has been constructed, one can compute the following numerical calibration metrics: ICI, E50, and E90. For each subject, we have a predicted probability of the cause-specific outcome occurring within time *t*. Then, using the smoothed calibration curve, one can determine an estimate of the smoothed observed probability of the cause-specific outcome occurring within time *t*. The ICI is computed as the mean absolute difference between observed and predicted probabilities across the sample. This is equivalent to the weighted absolute difference between the calibration curve and the diagonal line of best fit, where the difference is weighted by the distribution of predicted probabilities [11]. E50 is the median absolute difference between observed and predicted probabilities, while E90 is the 90th percentile of the absolute difference between observed and predicted probabilities. Let ${\hat{\mathrm{I}}}_{t_0}$denote the predicted probability of the occurrence of the outcome prior to time *t*_0_ and let ${{\hat{\mathrm{I}}}^s}_{t_0}$denote the smoothed or predicted probability based on the smoothed calibration curve (the latter is an estimate of the observed probability of the outcome that corresponds to the given predicted probability). The ${\mathrm{I}\mathrm{CI}}_{t_0}=\frac{1}{N}\sum \mid {\hat{\mathrm{I}}}_{t_0}^s-{\hat{\mathrm{I}}}_{t_0}\mid$, while $\mathrm{E}{50}_{t_0}$ is the median of $\mid {\hat{\mathrm{I}}}_{t_0}^s-{\hat{\mathrm{I}}}_{t_0}\mid$across the sample, and $\mathrm{E}{90}_{t_0}$ is the 90th percentile of $\mid {\hat{\mathrm{I}}}_{t_0}^s-{\hat{\mathrm{I}}}_{t_0}\mid$across the sample.

## Monte Carlo simulations: Methods

We conducted a sequence of six sets of Monte Carlo simulations to examine the ability of the method described above to assess the calibration of competing risk regression models. The first set of simulations examined the choice of the number of knots when using restricted cubic splines to estimate observed risk when constructing a smoothed calibration curve. The second set of simulations examined the performance of our proposed method when the fitted model was correctly specified and when censoring was present. The third set of simulations was a more extensive examination of the performance of the method when the model was correctly specified and censoring was absent (the absence of censoring allowed us to investigate a wider range of scenarios). The fourth set of simulations examined the performance of the method when the fitted model omitted a quadratic term. The fifth set of simulations examined the performance of the method when the fitted model omitted a main effect. The sixth set of simulations examined the performance of the method when the fitted model was correctly specified, but the CIF was different in the validation sample than in the derivation sample.

### Choice of number of knots for the restricted cubic spline model

The number of knots used in the restricted cubic splines when modelling the relationship between the subdistribution hazard of the cause-specific outcome (in the model used for estimating observed risk) and the predicted probability of the outcome (derived from the model whose calibration we want to assess) within a given duration of time can be thought of as a hyper-parameter. We conducted a series of simulations to determine the optimal number of knots when the underlying regression model was specified correctly.

We simulated data for a super-population consisting of 1,000,000 subjects. For each subject, we simulated a continuous covariate *x*, which can be thought of as a risk score, from a standard normal distribution: *x*~*N*(0, 1).

We assume that there are two types of events: type 1 events (the primary event of interest) and type 2 events (the competing event). When simulating event types and event times, we used a method of indirect simulation described by Beyersmann et al. [14] (Section 5.3.6), which in turn is based on an approach described by Fine and Gray [13]. This data-generating process is similar to that used in previous studies by the first author [15, 16]. In the data-generating process, the parameter *p* denotes the proportion of subjects with covariate equal to 0 who experience the event of interest as *t* → ∞. Furthermore, let *β*_1_ and *β*_2_ denote the logarithm of the subdistribution hazard ratios for the single covariate for the primary event and the competing event, respectively. Let *β*_1_*x* and *β*_2_*x* denote the linear predictor for the primary event and competing risk, respectively. An event type indicator, *Z*, was drawn from a Bernoulli distribution with subject-specific parameter $1-{\left(1-p\right)}^{\exp \left({\beta}_1x\right)}$ (*Z* = 1 primary event; *Z* = 0 competing event). The time of the competing event was drawn from an exponential distribution with rate parameter ${e}^{\beta_2x}$: ${T}_2\sim \exp \left({e}^{\beta_2x}\right)$. The time of the primary event was generated as ${T}_1=-\log \left(-\left(1-{\left(\left(-u+1/\varphi \right)\times \left(\varphi \right)\right)}^{1/{e}^{\beta_1x}}-p\right)/p\right)$, where $\varphi =1-{\left(1-p\right)}^{e^{\beta_1x}}$ and *u*~U(0, 1). The observed event time was determined as: *ZT*_1_ + (1 − *Z*)*T*_2_. For this set of simulations, *β*_1_ and *β*_2_ were fixed at 1 and 0.25, respectively.

We determined the 10th, 25th, 50th, 75th, and 90th percentiles of event times (regardless of the type of event) in the super-population. We refer to these times as *t*_10_, *t*_25_, *t*_50_, *t*_75_, and *t*_90_, respectively. These are the times at which we will assess calibration.

From the super-population, we drew a random sample of size 1000. In this sample, we used a Fine-Gray subdistribution hazards model to regress the subdistribution hazard of the outcome on the single covariate *X*. The calibration of the fitted model in the sample was assessed using restricted cubic splines with k knots, as described above. Smoothed calibration curves were constructed to evaluate the calibration of the fitted model at the five times described above: *t*_10_, *t*_25_, *t*_50_, *t*_75_, and *t*_90_. This process was repeated 1000 times and the mean calibration curve was estimated across the 1000 simulation replicates (the values of each of the 1000 calibration curves were evaluated along the same grid; for each value on that grid, we determined the mean value across the 1000 calibration curves). We considered three different values for the number of knots: 3, 4, and 5. The knots were located at specified percentiles in accordance with Harrell’s suggestion (i.e., for three knots, the locations were the 10th, 50th, and 90th percentiles) [1]. We allowed one factor to vary in the simulations: the parameter *p*. We allowed *p* to take on three different values: 0.25, 0.50, and 0.75.

### Correctly specified model in the presence of censoring

These simulations were similar to those described above with three exceptions. First, we fixed the number of knots for the restricted cubic spline model at three, based on the results from the previous set of simulations. Second, we introduced the presence of censoring and allowed the proportion of subjects who were censored to vary across scenarios. We allowed the proportion of subjects that were censored to range from 0 to 0.60 in increments of 0.20. Third, the sample size was fixed at 2000.

In order to incorporate censoring, we modified the data-generating process so that for each subject we simulated an event time (using methods identical to those described above) and a censoring time. Censoring times were simulated from an exponential distribution. For each subject, the observed survival time was the minimum of the simulated event time and the simulated censoring time. Subjects were considered censored observations if the censoring time was less than the event time. A bisection approach was used to determine the rate parameter for the exponential distribution so that the proportion of censored subjects in the super-population was equal to the desired value. Due to the presence of censoring, we evaluated calibration at the specified quantiles of the observed survival time in the large super-population, rather than at the specified quantiles of event times.

### Correctly specified model in the absence of censoring

This set of simulations was similar to those described in “Correctly-specified model in the presence of censoring”, except that we did not incorporate censoring. The rationale for this modification was that it allowed us to examine a wider variety of scenarios and to simplify the presentation of the results. We allowed the following two factors to vary (which had been fixed above): the size of the random samples (three values: 500, 1000, and 2000) and *β*_1_ (the subdistribution log-hazard ratio for the primary outcome) (three values: 0.25, 0.50, and 1). As above, *p* had three levels: 0.25, 0.50, and 0.75 and *β*_2_was fixed at 0.25. We thus considered 27 scenarios (3 sample sizes × 3 values of *β*_1_ × 3 values of *p*) in a full factorial design.

### Model with quadratic relationship

The simulations described above evaluated the performance of the graphical calibration methods when the survival model was correctly specified. This further set of simulations was similar to those described in “Correctly-specified model in the absence of censoring”, with the following modifications. Event times were simulated so that the linear predictor for the subdistribution hazard function for the primary outcome (*β*_1_*x* above) was replaced with *β*_1_*x* + 0.25*β*_1_*x*^2^. Thus, the logarithm of the subdistribution hazard of the primary outcome has a quadratic relationship with the continuous covariate *x*. In each random sample of size *N*, a mis-specified Fine-Gray subdistribution hazards model was fit. The model incorporated only a linear term for *x* and omitted the *x*^2^ term. We considered the same 27 different scenarios that were considered in “Correctly-specified model in the absence of censoring”. We did not incorporate censoring in this set of simulations as it was shown to have no effect in the previous set of simulations (“Correctly-specified model in the absence of censoring” vs “Correctly-specified model in the presence of censoring”) and to simplify the presentation of the results.

### Model with omitted main effect

This set of simulations was similar to those described in “Correctly-specified model in the absence of censoring”, with the following modifications. First, two covariates were simulated from a multivariate normal distribution. The mean of each covariate was equal to 0, while its variance was equal to 1. The correlation between the two covariates was set equal to *ρ* (see below). Event times were simulated so that the linear predictor for the subdistribution hazard function for the primary outcome (*β*_1_*x* above) was replaced with 0.50*x*_1_ + *β*_2_*x*_2_. Thus, the logarithm of the subdistribution hazard of the primary outcome was linearly related to each of the two covariates. Furthermore, the linear predictor for the subdistribution hazard function for the competing event was set equal to 0.25*x*_1_ + 0.25*x*_2_. In each random sample, a mis-specified Fine-Gray subdistribution hazards model was fit. The model incorporated only a linear term for *x*_1_ and omitted the *x*_2_ term. The size of each random sample was fixed at 1000, while the parameter p (the proportion of events that were type 1 events, as defined above) was fixed at 0.50. We allowed two factors to vary: *ρ* and *β*_2_. The correlation between the two covariates (*ρ*) took on four values: 0, 0.25, 0.50, and 0.75. The regression coefficient *β*_2_ took on 3 values: 0.25, 0.50, and 1. Using a full factorial design, we thus considered 12 different scenarios. We did not incorporate censoring in this set of simulations as it was shown to have no effect in a previous set of simulations and to simplify the presentation of the results.

### The incidence of the outcome differs between the derivation and validation samples

This set of simulations was similar to those described in “Correctly-specified model in the absence of censoring”, with the following modifications. Rather than simulate one super-population, we simulated two super-populations, such that the distribution of the baseline covariate was the same in each of the two super-populations. We fixed the following factors: *β*_1_, the subdistribution log-hazard ratio for the primary outcome, was fixed at 0.50, and *p* was fixed at 0.50, while *β*_2_ was fixed at 0.25. Outcomes were generated in the first super-population using the data-generating process described above. In the second super-population, we simulated outcomes, not using *p* = 0.50, but using *p* = *p*.validation (see below for values). We drew a random sample of size 1000 from the first super-population and a random sample of the same size from the second super-population. These will serve as the derivation and validation samples, respectively. The correctly specified model was estimated in the derivation sample and was then applied to the validation sample, where its calibration assessed. The factor *p*.validation was allowed to take five different values: 0.3, 0.4, 0.5, 0.6, and 0.7 (*p* = 0.50 was included as a control to provide a benchmark against to which compare different values of ICI, E50, and E90). Thus, we considered scenarios in which the proportion of subjects with covariate equal to 0 who experience the event of interest as *t* → ∞ differs between the derivation and validation samples.

### Software

In these simulations, the subdistribution hazard models were fit using the FGR function in the riskRegression package (version 2019.11.03) for R (version 3.5.1). Restricted cubic splines were implemented using the rcspline.eval function from the rms package (version 5.1-4) for R.

## Monte Carlo simulations: Results

In presenting the results of the simulations, we present the results for the scenarios with *p* = 0.50 in the manuscript and the results for the scenarios with *p* = 0.25 and 0.75 in the supplemental online appendix. In most instances, results did not differ meaningfully between scenarios with *p*= 0.50 and scenarios with *p* = 0.25 or 0.75.

### Number of knots for the restricted cubic splines

**Fig. 1:**
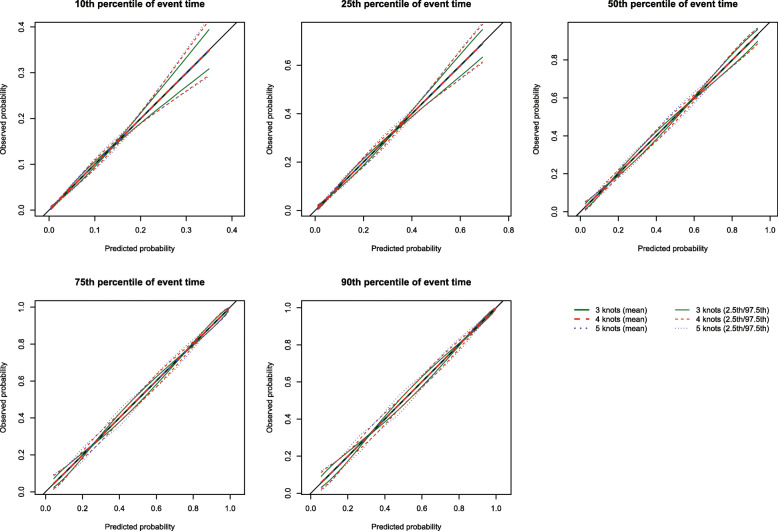
RCS: Choice of number of knots (*p* = 0.50)

The mean estimated calibration curves across the 1000 simulation replicates are described in Fig. 1 for *p* = 0.50 and in Figs. A1 and A2, in the supplemental online appendix, for *p* = 0.25 and 0.75. Each figure consists of five panels, one for each of the five time points at which calibration was assessed (*t*_10_, *t*_25_, *t*_50_, *t*_75_, and *t*_90_). Each panel displays the mean calibration curve for each of the three values of the number of knots (3, 4, and 5 knots). For each value on the grid of predicted probabilities along which the mean calibration curve was estimated (see above), we also estimated the 2.5th and 97.5th percentiles of the observed probabilities across the 1000 sampled datasets. Using these estimated percentiles, we have superimposed curves for each of the three values of the number of knots reflecting the variability in the estimated calibration curve across the 1000 simulation replicates. Across the five times at which we assessed calibration and the three values of *p*, the choice of knot (3 vs. 4 vs. 5) did not have an effect on the mean calibration curve. For all choices of knots, the mean calibration curve coincided with the diagonal line denoting perfect calibration. However, the estimated calibration curves displayed slightly increasing variability across simulation replicates as the number of knots increased from three to five.

**Fig. 2:**
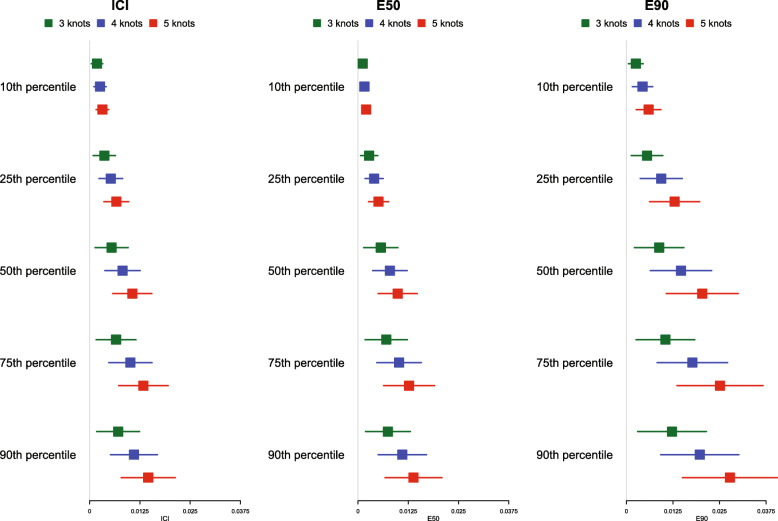
ICI/E50/E90 in simulations for selecting the optimal number of knots (*p* = 0.50)

The mean estimated values of the ICI, E50, and E90, along with their standard deviation (the standard deviations of the different calibration metrics are reported as error bars) across the 1000 simulated samples are reported in Fig. 2 for *p* = 0.50 and in Figs. A3 and A4 in the online supplemental appendix for *p* = 0.25 and 0.75. Since a correctly specified model was fit, we want the values of ICI, E50, and E90 to be close to 0. For all combinations of time points (*t*_10_, *t*_25_, *t*_50_, *t*_75,_ and *t*_90_), values of *p*, and metrics (ICI, E50, and E90), mean calibration was better when three knots were used than when four or five knots were used. Furthermore, differences between the choice of number of knots increased as the quantile of survival time increased (e.g., differences between ICI for different numbers of knots was marginal for *t*_10_, while it was larger for *t*_90_).

Based on the results of these simulations, we concluded that the use of three knots is preferable to the use of four or five knots when using restricted cubic splines to compute calibration curves. Accordingly, this value was used in all subsequent simulations. Note that the optimal number of knots may vary depending on the complexity of the calibration curve, that is the complexity of the pattern of mis-calibration.

### Correctly specified regression model in the presence of censoring

**Fig. 3:**
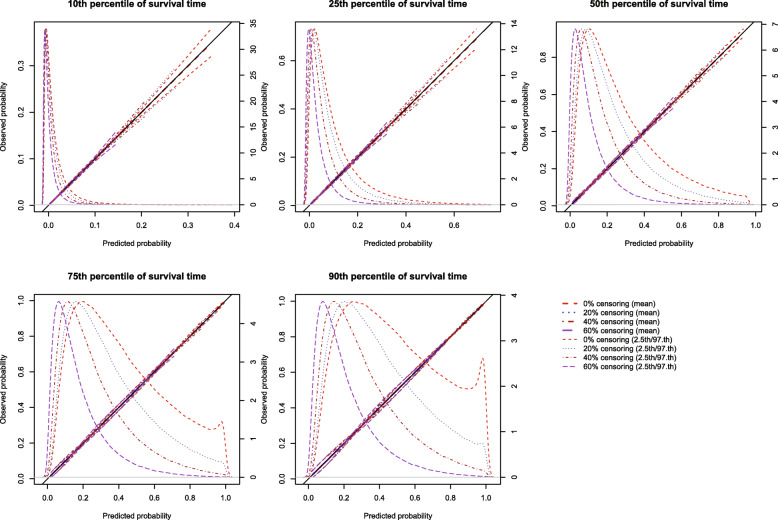
Effect of degree of censoring on estimated calibration curves (*N* = 2000 and *p* = 0.5)

The mean estimated calibration curves across the 1000 simulation replicates are described in Fig. 3 for *p* = 0.50 and in Figs. A5 and A6 for *p* = 0.25 and 0.75. Each calibration curve is restricted to a range of predicted probabilities ranging from the 1^st^ to the 99^th^ percentiles of risk in the population. Each figure consists of five panels, one for each of the five time points at which calibration was assessed (*t*_10_, *t*_25_, *t*_50_, *t*_75,_ and *t*_90_). Each panel depicts the mean calibration curve for the given method of constructing calibration curves, along with lines denoting the 2.5th and 97.5th percentiles of the calibration curves across the 1000 simulation replicates. This pair of curves provides an assessment of the variability of the calibration curves across simulation replicates. There is one set of curves for each of the different degrees of censoring. On each panel, we have superimposed a diagonal line denoting perfect calibration. On each panel, we have also superimposed non-parametric estimates of the density of the predicted probabilities in the large super-population (right vertical axis). Note that there is a separate density function for each of the different degrees of censoring (note that the density function differs across the different degrees of censoring because the time points at which calibration is assessed differ across these scenarios).

Regardless of the degree of censoring, our proposed method resulted in calibration curves that were close to the diagonal line of perfect calibration over the range of predicted probabilities in which most subjects lay.

**Fig. 4:**
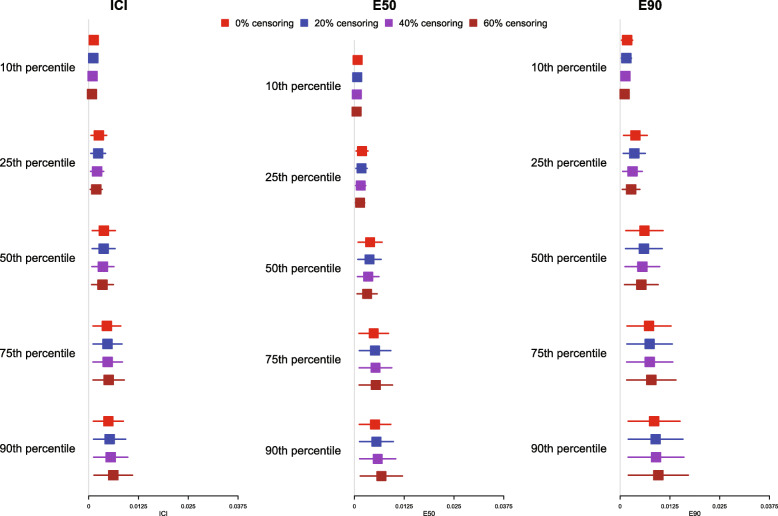
ICI/E90/E90 for correctly specified model and censoring (*N* = 2000 and *p* = 0.50)

The mean estimated values of calibration metrics are reported in Fig. 4 for *p* = 0.50 and in Figs. A7 and A8 for *p* = 0.25 and 0.75. Since the fitted model was correctly specified, we want the values of the calibration metrics to be close to 0. Each metric tended to be close to 0 for most settings and times at which calibration was assessed. For each metric, the mean estimated metric tended to increase as the percentile of survival time at which calibration was assessed increased. Furthermore, the estimated metrics displayed greater variability across simulation replicates as the percentile of survival time at which calibration was assessed increased.

### Correctly specified regression model in the absence of censoring

**Fig. 5:**
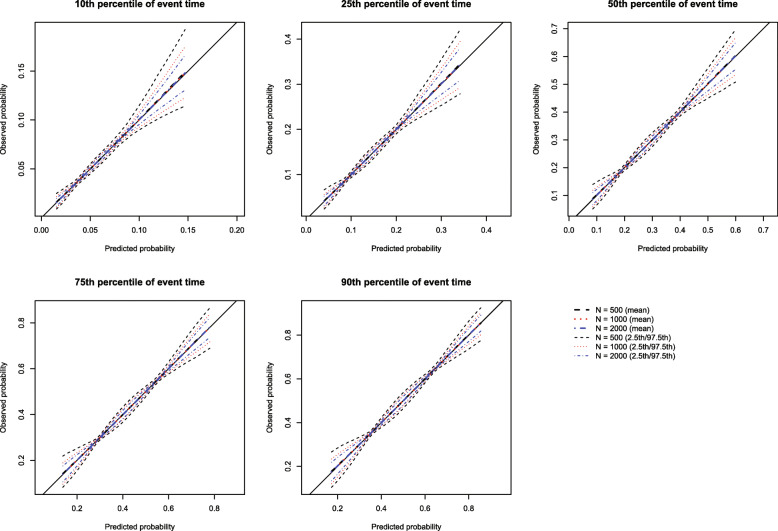
True model fitted with no censoring (*β*_1_ = 0.50 and *p* = 0.50)

The mean estimated calibration curves across the 1000 simulation replicates are described in Fig. 5 for the scenario with *β*_primary_ = 0.50 and *p* = 0.50. Results for the other eight combinations of these two factors are summarized in Figs. A9 to A16. Each figure consists of five panels, one for each of the five percentiles of survival time at which calibration was assessed. In each panel, we report the mean estimated calibration curve (along with confidence limits) for the scenarios with *N* = 500, 1000, and 2000. In 26 of the 27 scenarios, the mean calibration curve coincided with the diagonal line denoting perfect calibration. The one exception (Fig. A9) occurred with *N* = 500, *β*_primary_ = 0.25, and *p* = 0.25 when assessing calibration at the 10th and 25th percentiles of event time. In this one setting, minor deviation from the line of perfect calibration was assessed. We suspect that this one deviation is due to the low number of primary events that would be observed in this scenario. Across the scenarios, the estimated calibration curves displayed less variability across simulation replicates as the sample size increased.

**Fig. 6:**
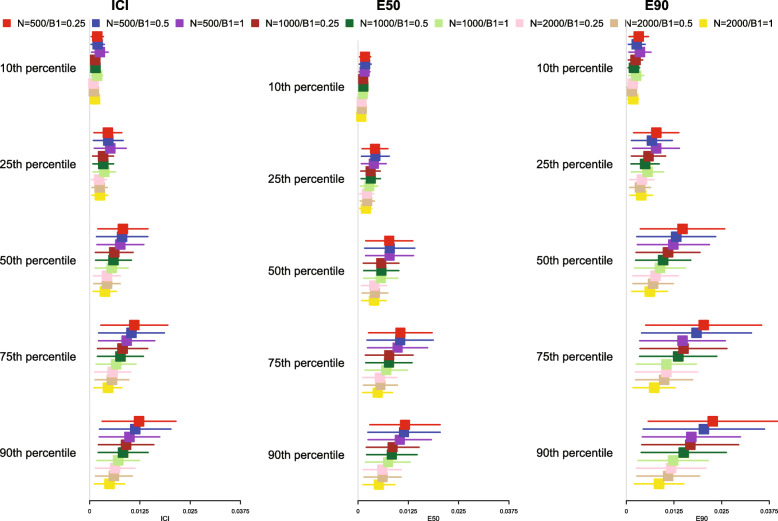
ICI/E90/E90 for correctly specified model without censoring (*p* = 0.50)

The mean estimated calibration metrics (along with their standard deviations across the simulation replicates) are reported in Fig. 6 for *p* = 0.50 and Figs. A17 and A18 for *p* = 0.25 and 0.75. Since the correctly specified model was fit, we want the calibration metrics to be close to 0. We observe that as sample size increases and as *β*_primary_ increases, the mean estimated metric tended to become closer to 0. Each calibration metric diverged from 0 and displayed more variability as the time at which calibration was assessed increased.

### Incorrectly specified regression model: omission of a quadratic term

**Fig. 7:**
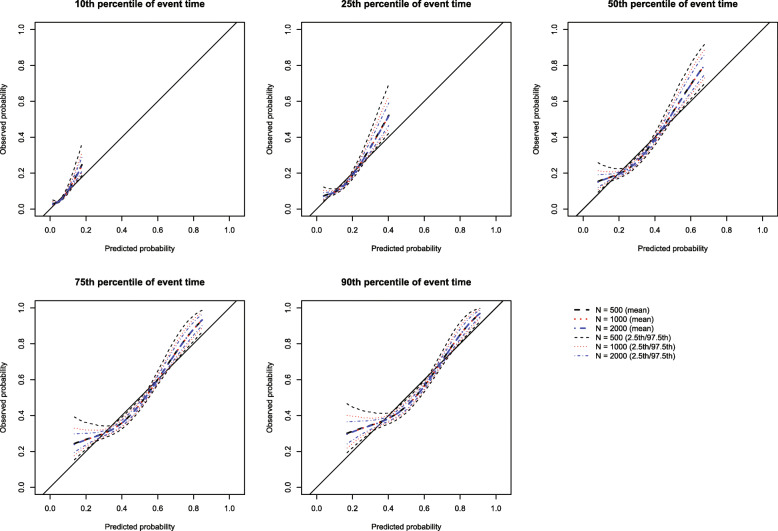
Mis-specified model (*β*_1_ = 0.50 and *p* = 0.50)

The mean estimated calibration curves across the 1000 simulation replicates are described in Fig. 7 for *β*_primary_ = 0.5 and *p* = 0.50. Results for the other eight combinations of these two factors are summarized in Figs. A19 to A26. The figures have a similar structure to those described in “Correctly-specified regression model in the absence of censoring”. In all figures, the mean calibration curves differed from the diagonal line of perfect calibration. The mean calibration curves tended to have an approximately quadratic shape, providing evidence that a quadratic term had been omitted from the model. The variation displayed by the calibration curves decreased with increasing sample size.

**Fig. 8:**
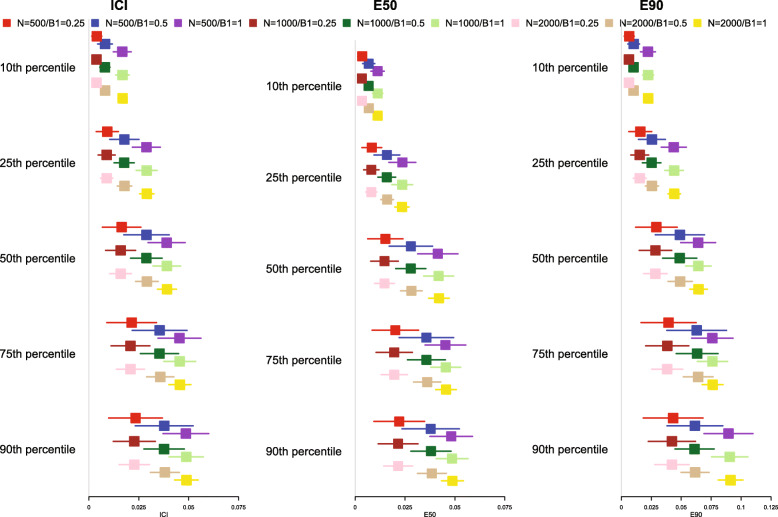
ICI/E90/E90 for incorrectly specified model (*p* = 0.50)

The mean estimated values of the ICI, E50, and E90 are reported in Fig. 8 for *p* = 0.50 and in Figs. A27 and A28 for *p* = 0.25 and 0.75. Since the quadratic term was omitted from the model, we want the values of the calibration metrics to be different from 0, indicating that the models are miscalibrated. For a given sample size, we observe that the mean calibration metric (ICI, E50, and E90) tended to increase as the value of *β*_primary_ increased. As expected, the variability of the estimated metric across simulation replicates decreased with increasing sample size. In comparing this set of figures with the corresponding set of figures for when the correctly specified model was fit, we observe that, across all 27 scenarios and 5 percentiles of survival time, the estimated metrics were larger when the incorrectly specified model was fit compared to when the correctly specified model was fit.

### Incorrectly specified regression model: omission of a main effect

**Fig. 9:**
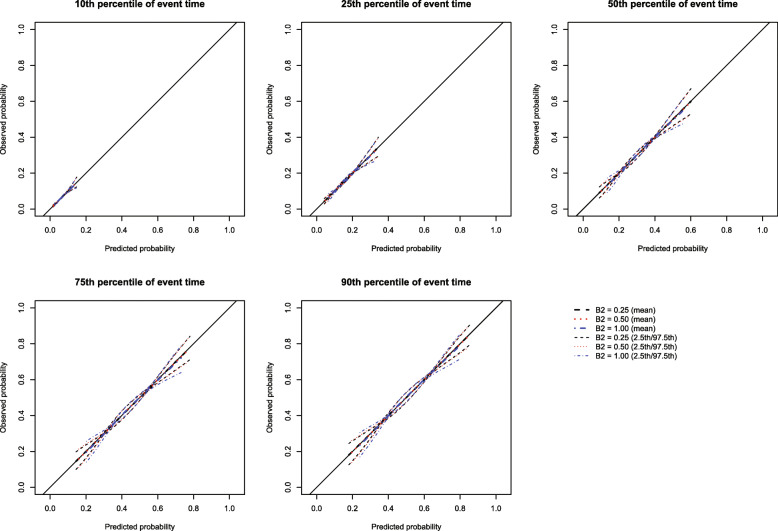
Mis-specified model (omission of main effect) (*ρ* = 0)

The mean estimated calibration curves across the 1000 simulation replicates are described in Fig. 9 for the three scenarios involving *ρ* = 0. Results for the other 9 combinations of the two factors are summarized in Figs. A29 to A31. In all figures, the mean calibration curves did not differ from the diagonal line of perfect calibration.

**Fig. 10:**
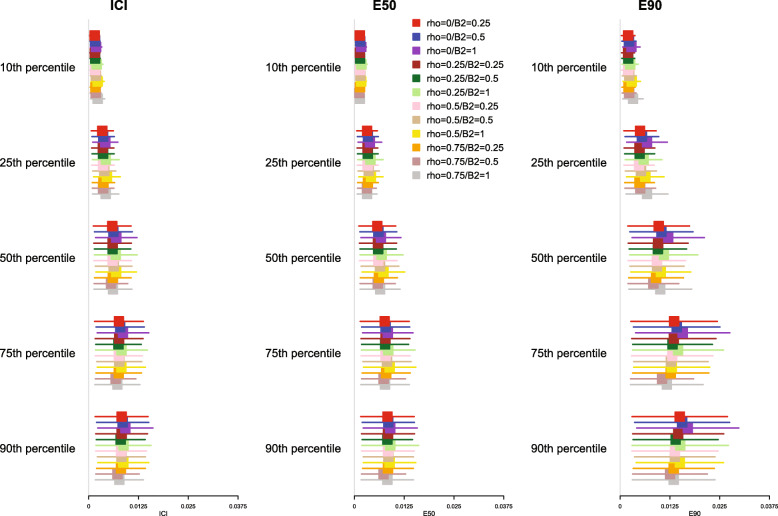
ICI/E90/E90 for incorrectly specified model (omission of main effect)

The mean estimated values of the ICI, E50, and E90 are reported in Fig. 10 for all 12 scenarios. For all calibration metrics (ICI, E50, and E90), and for a given time at which calibration was assessed, the value of the metric did not vary across the 12 different scenarios. Across all 12 scenarios and all five times at which calibration was assessed, the mean calibration metric was not meaningfully different from 0 and therefore did not identify miscalibration due to the omission of a main effect. For a given scenario and metric, the mean metric increased as the time at which calibration was assessed increased.

### The incidence of the outcome differs between the derivation and validation samples

**Fig. 11:**
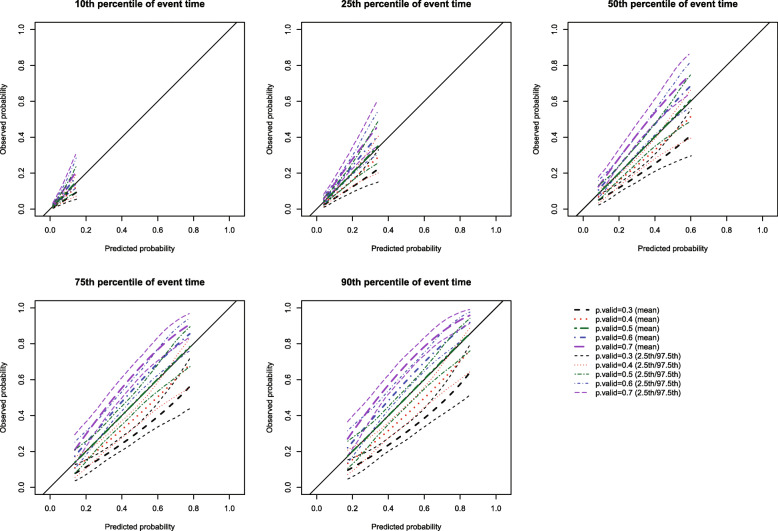
*p* different in validation sample (*p* = 0.50)

The mean estimated calibration curves across the 1000 simulation replicates are described in Fig. 11 for all five scenarios. Across all four scenarios in which *p*.valid was different from 0.50 and across all five times at which calibration was assessed, the mean calibration curve deviated from the diagonal line of perfect calibration. In contrast, as expected, when the parameter p was the same in both populations, the mean calibration curve did not deviate from the line of perfect calibration.

**Fig. 12:**
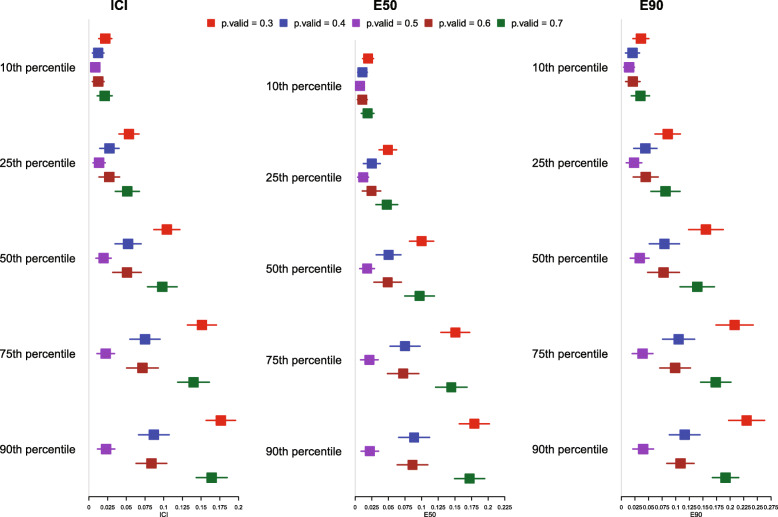
ICI/E90/E90 when *p* is different in validation sample

The mean estimated values of the ICI, E50, and E90 are reported in Fig. 12 for all five scenarios. For all calibration metrics (ICI, E50, and E90), and for a given time at which calibration was assessed, the value of the metric increased. As *p*.valid diverged from 0.50, the value of each of the three metrics increased compared to that observed when the value of *p*.valid was 0.50 (the value in the derivation population).

## Case study

We provide a case study to illustrate the utility of graphical methods for assessing the calibration of competing risk regression models. In the biomedical literature, there is an increasing interest in comparing the performance of conventional statistical methods with machine learning methods for predicting outcomes for patients with cardiovascular disease [17–20]. Given this interest, we compare the calibration of a Fine-Gray proportional subdistribution hazard model (a conventional statistical model) with that of a random survival forest for competing risk data (a machine learning method) for predicting the incidence of cardiovascular mortality. We assess the calibration of predictions of the probability of death within 1, 2, 3, 4, and 5 years using each approach.

In the current case study, we focus only on assessing the calibration of models for predicting the incidence of cardiovascular death. Predicting the incidence of cardiovascular death is important as it can inform medical decision. However, the risk of all-cause mortality is also important from a patient’s perspective. Thus, knowledge of both the risk of all-cause mortality and cause-specific mortality is important. The calibration of models for all-cause mortality can be assessed using the methods described above. However, this is not the focus of the current case study.

### Data sources

The Enhanced Feedback for Effective Cardiac Treatment (EFFECT) Study was an initiative to improve the quality of care for patients with cardiovascular disease in Ontario [21]. During the first phase, detailed clinical data were collected on patients hospitalized with congestive heart failure (CHF) between April 1, 1999, and March 31, 2001, at 86 hospital corporations in Ontario, Canada. During the second phase, data were abstracted on patients hospitalized with this condition between April 1, 2004, and March 31, 2005, at 81 Ontario hospital corporations. Data on patient demographics, vital signs and physical examination at presentation, medical history, and results of laboratory tests were collected for these two samples. In this case study, the first phase of the EFFECT sample will be used for model derivation, while the second phase will be used as an independent validation sample from a different temporal period. For the current case study, 8238 and 7608 subjects were available from the first and second phases, respectively. By using an independent validation sample from a different era we will be assessing external validation of the estimated prediction models. This is distinct from internal validation, in which the performance of a model is assessed in the sample in which it was derived (or which is statistically identical to that in which it was developed).

The outcome for the case study was time from hospital admission to cardiovascular death, with subjects censored after 5 years of follow-up if death had not yet occurred. Death due to non-cardiovascular causes was treated as a competing risk. In the first phase of the study, 41.5% and 27.1% of patients died of cardiovascular and non-cardiovascular causes, respectively, within 5 years of hospital admission. In the second phase of the study, 37.2% and 30.2% of patients died of cardiovascular and non-cardiovascular causes, respectively, within 5 years of hospital admission. Of note, for interpreting subsequent results, the 5-year incidence of cardiovascular death decreased, while that of non-cardiovascular death increased between the two studies periods.

### Methods

The candidate predictor variables considered in this case study were: age, sex, systolic blood pressure, heart rate, respiratory rate, neck vein distension, S3, S4, rales > 50% of lung field, pulmonary edema, cardiomegaly, diabetes, cerebrovascular disease/transient ischemic attack, previous acute myocardial infarction, atrial fibrillation, peripheral vascular disease, chronic obstructive pulmonary disease, dementia, cirrhosis, cancer, left bundle branch block, haemoglobin, white blood count, sodium, potassium, glucose, urea, and creatinine.

We fit a Fine-Gray proportional subdistribution hazard model in the derivation sample (EFFECT phase 1) in which the subdistribution hazard of cardiovascular mortality was regressed on all the variables listed above. The fitted model was then applied to the independent validation sample (EFFECT phase 2). We also fit two random survival forests, each of which accounted for the competing risk of non-cardiovascular death, in the derivation sample, in which the hazard of cardiovascular mortality was modelled using the covariates listed above [22]. For each random survival forest, 1000 survival trees were grown. In the first random forest, we used the default value for the node size parameter (forest average number of unique cases in a terminal node; default = 15). In the second random forest, we used fivefold cross-validation in the derivation sample to determine the optimal node size. The optimal node size was 150, when using ICI at 1 year in the derivation sample as the optimization criterion. This latter analysis illustrates the utility of a numerical calibration metric for use in tuning machine learning methods. The fitted survival forests were then applied to the independent validation sample. We evaluated the calibration of these methods in the validation sample at 1, 2, 3, 4, and 5 years post-admission. Graphical calibration curves were computed, as were ICI, E50, and E90. Calibration metrics were estimated using the crprep function from the mstate package (version 0.2.11) for R combined with the cph function from the rms package (version 5.1-4) [23]. R code for conducting these analyses is provided in the Appendix.

### Results

**Fig. 13:**
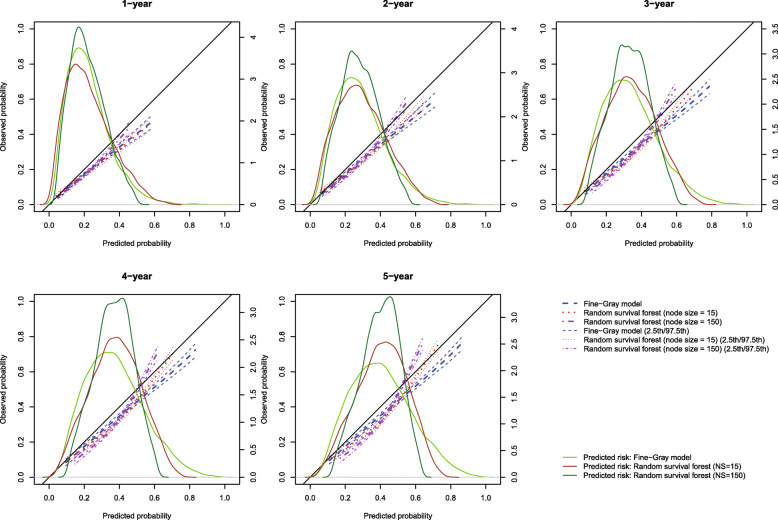
Calibration plots (predicting cardiovascular death) for Fine-Gray model and random survival forests for case study

The calibration plots for the two methods of estimating the risk of cardiovascular death are described in Fig. 13. This figure consists of five panels, one each for assessing calibration at 1, 2, 3, 4, and 5 years post-admission. As in the previous figures, we have assessed calibration over an interval ranging from the 1st percentile of predicted probabilities to the 99th percentile of predicted probabilities. On each panel, we superimposed the density functions for the predicted probabilities of death as derived from each of the three prediction models. The distribution of predicted risks from the Fine-Gray model was more right-skewed than the distribution arising from the random survival forest with node size equal to 150, at each of the five times at which calibration was assessed. Thus, there were more subjects who had large predicted probabilities arising from the Fine-Gray model than from the random survival forest.

In examining Fig. 13, one observes that, when predicting the incidence of cardiovascular death within 1 and 2 years, the Fine-Gray model and the random survival forest with the optimal node size (150) had comparable calibration when the predicted probability of cardiovascular death was less than 0.30. For predicted values above this level, the random survival forest tended to have better calibration. When predicting incidence within 3, 4, and 5 years, the Fine-Gray model tended to display better calibration when the predicted incidence was less than 0.40, while the random survival forest displayed better calibration when the predicted incidence was above 0.40. In general, for both methods, predicted incidence tended to be higher than observed incidence. This overprediction is not surprising, given that the incidence of cardiovascular death was higher in the phase 1 sample than in the phase 2 sample.

**Fig. 14:**
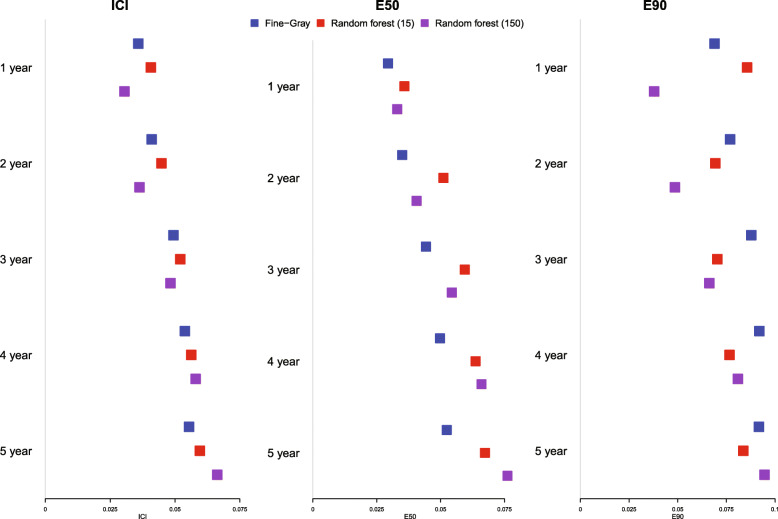
ICI/E50/E90 in case study validation sample (predicting cardiovascular mortality)

The estimated ICI, E50, and E90 are reported in Fig. 14. ICI was closer to 0 for the random forest with optimal node size than for the Fine-Gray model for predicting incidence at 1, 2, and 3 years, while the converse was true for predicting incidence at 4 and 5 years. E50 was lower for the Fine-Gray model than for the random forest at all five time points, while the converse was true for E90 at four of the five time points. This suggests that the “central miscalibration” (median of the absolute difference between predicted outcome risk and observed outcome risk) was smaller (better) for the FG model while the extremes of miscalibration (90th percentile of the absolute difference between predicted outcome risk and observed outcome risk) were smaller for the random forest. The extremes of miscalibration were also influenced by more heterogeneous risk predictions of the FG model, potentially leading to more pronounced miscalibration at the extremes of the predicted risk distribution (density plots of Fig. 13). The lack of agreement between the different metrics suggests that reporting all three metrics will allow for a more comprehensive comparison of the calibration of different models.

In comparing the calibration of the two random survival forests in the validation sample, we observe a substantial improvement in calibration for models in which the node size was tuned in the derivation sample compared to when the forest was grown using default settings. This illustrates the usefulness of a numeric calibration metric when tuning parameters of machine learning methods.

**Fig. 15:**
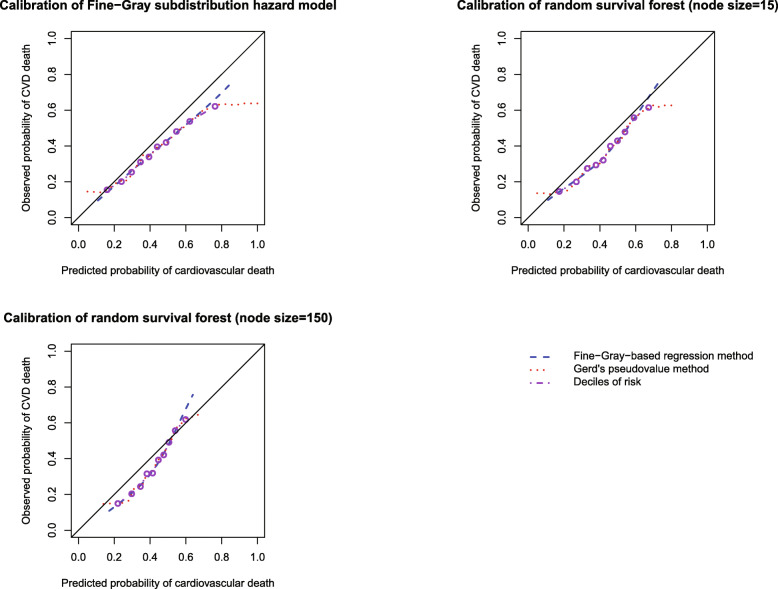
Comparison of different methods for assessing calibration (of predictions cardiovascular death) at 5 years

For comparative purposes, we compared our proposed methods for assessing calibration with two other approaches. As comparators, we considered the method described by Wolbers and colleagues, in which subjects are categorized according to the deciles of predicted risk [7]. Then, within each of the 10 risk strata, we compared the mean predicted risk and the empirically observed risk. We also used the method proposed by Gerds and colleagues based on jackknife pseudo-values combined with a nearest neighbour smoother [8]. We restricted our comparison of the different methods for assessing calibration to assessing calibration at 5 years. We assessed the calibration of the Fine-Gray subdistribution hazard model and the two random survival forests using the three different approaches. The comparison of methods for assessing calibration is reported in Fig. 15. There is one panel for each of the three prediction methods. The method based on the use of risk strata was unable to assess calibration at the extremes of predicted risk, as it was based on a categorization of risk. The three methods tended to result in a similar assessment of calibration for non-extreme values of predicted risk. Our proposed method and that of Wolbers and colleagues tended to disagree at the extremes of predicted risk. However, the direction of disagreement was not consistent. For example, our proposed method showed that the Fine-Gray model had better calibration at the extremes of predicted risk than did the assessment using Wolber’s method. However, Wolber’s method showed that the random survival forest with node size = 150 had better calibration at the extremes of predicted risk than did the assessment using our proposed method. However, apart from these disagreements at the extremes of predicted risk (where there are few subjects—see Fig. 13), the two methods produced comparable assessments.

## Conclusion

We described a method for constructing smoothed calibration curves for assessing the calibration of models for time-to-event outcomes in the presence of competing risks. From these smoothed calibration curves, three different numerical calibration metrics can be derived that quantify differences between predicted and observed probabilities. The use of these graphical calibration curves allows for an assessment of the calibration of competing risk models. The numeric calibration metrics will facilitate the comparison of the calibration of different models for competing risk data. Furthermore, as was done in our case study, the numeric calibration metrics can be used as optimization parameters when tuning machine learning methods.

Assessing calibration of prediction models is most useful in the context of external validation. The case study illustrated the usefulness of our framework when assessing model calibration in independent datasets. The framework may also be useful when assessing calibration of a model in the same data that was used for development of the model (apparent validation), because it identifies incorrect specification of, especially continuous, predictors. When the framework is used for assessing calibration as part of an internal validation—testing and validating the model in disjoint random subsets of the original data—the emphasis will be on understanding the amount of overfitting of the model, and on the need for parameter shrinkage.

Calibration is one aspect of assessing the accuracy of prediction models. Another important aspect of assessing the accuracy of prediction models is assessing discrimination. Methods for assessing the discrimination of prediction models for competing risks have been described elsewhere [24]. There is an extensive literature on assessing calibration of models for binary outcomes and survival outcomes in the absence of competing risks [1–5, 12, 25]. In clinical and epidemiological research, there is an increasing awareness of the importance of accounting for competing risks when analysing survival outcomes. As noted in the Introduction, only two prior studies have proposed for assessing the calibration of competing risk regression [7, 8]. An advantage of our proposed approach over the first method is that it avoids the categorization of predicted risk, which can be subjective and can lead to the loss of information. A further advantage of our approach is that is a natural extension to previously described methods for constructing smoothed calibration curves for binary outcomes or survival outcomes in the absence of competing risks [1–3, 5, 12].

We have described how to assess calibration of competing risk models using the Fine-Gray subdistribution hazard model to estimate observed risk at each level of predicted risk, thereby permitting construction of smoothed calibration curves. We focused on the use of the Fine-Gray model for estimating observed risk when constructing smoothed calibration curves, as it is the method most frequently used in the biomedical literature for estimating incidence in the presence of competing risks. However, alternatives exist to the Fine-Gray model, including fitting separate cause-specific hazard models for each of the different types of outcomes and then estimating the CIF by combining the individual estimated cause-specific hazard functions [10]. Our approach to assessing calibration does not require the use of the Fine-Gray model. Instead, any method that allows for estimating the CIF as a function of a continuous covariate can be used. We have recently shown that the use of the Fine-Gray model can have undesirable effects when one wants to estimate the incidence of all of the different competing events [26]. In such settings, the use of the separate cause-specific hazard models may be preferable. Our primary rationale for focusing on the subdistribution hazard model in the current application was due to it being the most popular method to estimate the CIF in the presence of competing risks and we were interested in a single outcome type, and not all of the cause-specific outcomes.

There are certain limitations to the current study. Our evaluation of graphical calibration curves and calibration metrics for use with competing risk regression models was based on Monte Carlo simulations. Due to the computational intensity of these simulations, we were only able to examine a limited number of scenarios. These simulations were not intended to be comprehensive. Instead, we illustrated that the calibration curves performed as intended when the model was correctly specified and that they were able to identify mis-specification due to omission of a quadratic term. Furthermore, they were able to identify miscalibration when the incidence of the outcome differed in the validation sample than in the derivation sample. However, they did not identify model mis-specification due to omission of a main effect. However, rather than concluding that our metrics are insensitive to this form of mis-specification, we argue that omission of a main effect from the model—a typical phenomenon in practice—does not necessarily lead to substantial miscalibration when the model is validated in a sample with the same covariate distribution. We provide a simple example for why this may be true. Consider a scenario in which the effect of age differs by sex. If sex is omitted from the prognostic model, we will estimate a weighted age effect in the derivation sample. These age effects may be well calibrated in a population with the same sex distribution but will be miscalibrated in populations with different sex distributions. In external validation samples with different covariate distributions—as is commonly observed in practice, and was also observed in the case study—our framework does identify miscalibration. While we examined a large number of scenarios in our Monte Carlo simulations, there are scenarios that were not examined.

The focus of the current study was on methods to assess the calibration of a prognostic model for use in the presence of competing risks (e.g., a Fine-Gray subdistribution hazard model or a method based on combining the cause-specific hazard functions). We have not addressed the issue of assessing whether the proportional hazards assumption holds for the prognostic model. However, our proposed method of estimating observed risk uses a Fine-Gray subdistribution hazard model, which requires the proportional hazard assumption. The impact of the violation of the proportional hazards assumption for the model used to estimate observed risk when constructing smooth calibration curves is not clear. For the Cox model in standard survival analysis in the absence of competing risks, if one applies administrative censoring at the prediction horizon (the time at which one wants to estimate survival probabilities) then, in the presence of non-proportional hazards, the predictions based on the Cox model at that prediction horizon will be approximately correct [27, 28]. While it is not known if this is true for the Fine-Gray model, we speculate that the same result will hold and that it might be advisable to apply administrative censoring when assessing calibration in the presence of competing risks if one suspects that the proportional hazards assumption is violated for the model used for estimating observed risk.

In summary, we have described and evaluated a method for constructing calibration curves and numerical calibration metrics for models for time-to-event outcomes in the presence of competing risks. The use of graphical calibration curves constructed using Fine-Gray subdistribution hazard models allows for an assessment of the calibration of competing risk models. Using this approach, the calibration of any model for estimating incidence in the presence of competing risks, and not just the Fine-Gray model, can be assessed. The numeric calibration metrics will facilitate the comparison of the calibration of different models for competing risk data.

## Supplementary Information

**Additional file 1.** **Figure S1.** RCS: Choice of number of knots (p = 0.25). **Figure S2.** RCS: Choice of number of knots (p = 0.75). **Figure S3.** ICI/E50/E90 in simulations for selecting the optimal number of knots (p = 0.25). **Figure S4.** ICI/E50/E90 in simulations for selecting the optimal number of knots (p = 0.75). **Figure S5.** Effect of degree of censoring on estimated calibration curves (N = 2000 and p = 0.25). **Figure S6.** Effect of degree of censoring on estimated calibration curves (N = 2000 and p = 0.75). **Figure S7.** ICI/E90/E90 for correctly−specified model and censoring (N = 2000 and p = 0.25). **Figure S8.** ICI/E90/E90 for correctly−specified model and censoring (N = 2000 and p = 0.75). **Figure S9.** True model fitted with no censoring (beta1 = 0.25 & p = 0.25). **Figure S10.** True model fitted with no censoring (beta1 = 0.25 & p = 0.50). **Figure S11.** True model fitted with no censoring (beta1 = 0.25 & p = 0.75). **Figure S12.** True model fitted with no censoring (beta1 = 0.50 & p = 0.25). **Figure S13.** True model fitted with no censoring (beta1 = 0.50 & p = 0.75). **Figure S14.** True model fitted with no censoring (beta1 = 1 & p = 0.25). **Figure S15.** True model fitted with no censoring (beta1 = 1 & p = 0.50). **Figure S16.** True model fitted with no censoring (beta1 = 1 & p = 0.75). **Figure S17.** ICI/E90/E90 for correctly−specified model without censoring (p = 0.25). **Figure S18.** ICI/E90/E90 for correctly−specified model without censoring (p = 0.75). **Figure S19.** Mis−specified model (beta1 = 0.25 & p = 0.25). **Figure S20.** Mis−specified model (beta1 = 0.25 & p = 0.50). **Figure S21.** Mis−specified model (beta1 = 0.25 & p = 0.75). **Figure S22.** Mis−specified model (beta1 = 0.50 &amp; p = 0.25). **Figure S23.** Mis−specified model (beta1 = 0.50 & p = 0.75). **Figure S24.** Mis−specified model (beta1 = 1 & p = 0.25). **Figure S25.** Mis−specified model (beta1 = 1 & p = 0.50). **Figure S26.** Mis−specified model (beta1 = 1 & p = 0.75). Figure S27. ICI/E90/E90 for incorrectly−specified model (p = 0.25). **Figure S28.** ICI/E90/E90 for incorrectly−specified model (p = 0.75). **Figure S29.** Mis−specified model (omission of main effect) (rho=0.25). **Figure S30.** Mis−specified model (omission of main effect) (rho=0.50). **Figure S31.** Mis−specified model (omission of main effect) (rho=0.75). :R code for constructing calibration curves and numerical metrics of calibration using restricted cubic splines.

## Data Availability

The data sets used for this study were held securely in a linked, de-identified form and analysed at ICES. While data sharing agreements prohibit ICES from making the data set publicly available, access may be granted to those who meet pre-specified criteria for confidential access, available at www.ices.on.ca/DAS.
