# Supplementary material for: Graphical calibration curves and the integrated calibration index (ICI) for competing risk models
Source: Diagn Progn Res. 2022 Jan 17;6:2. doi: 10.1186/s41512-021-00114-6 (PMC8762819; doi:10.1186/s41512-021-00114-6)

Figure A1. RCS: Choice of number of knots (p = 0.25)

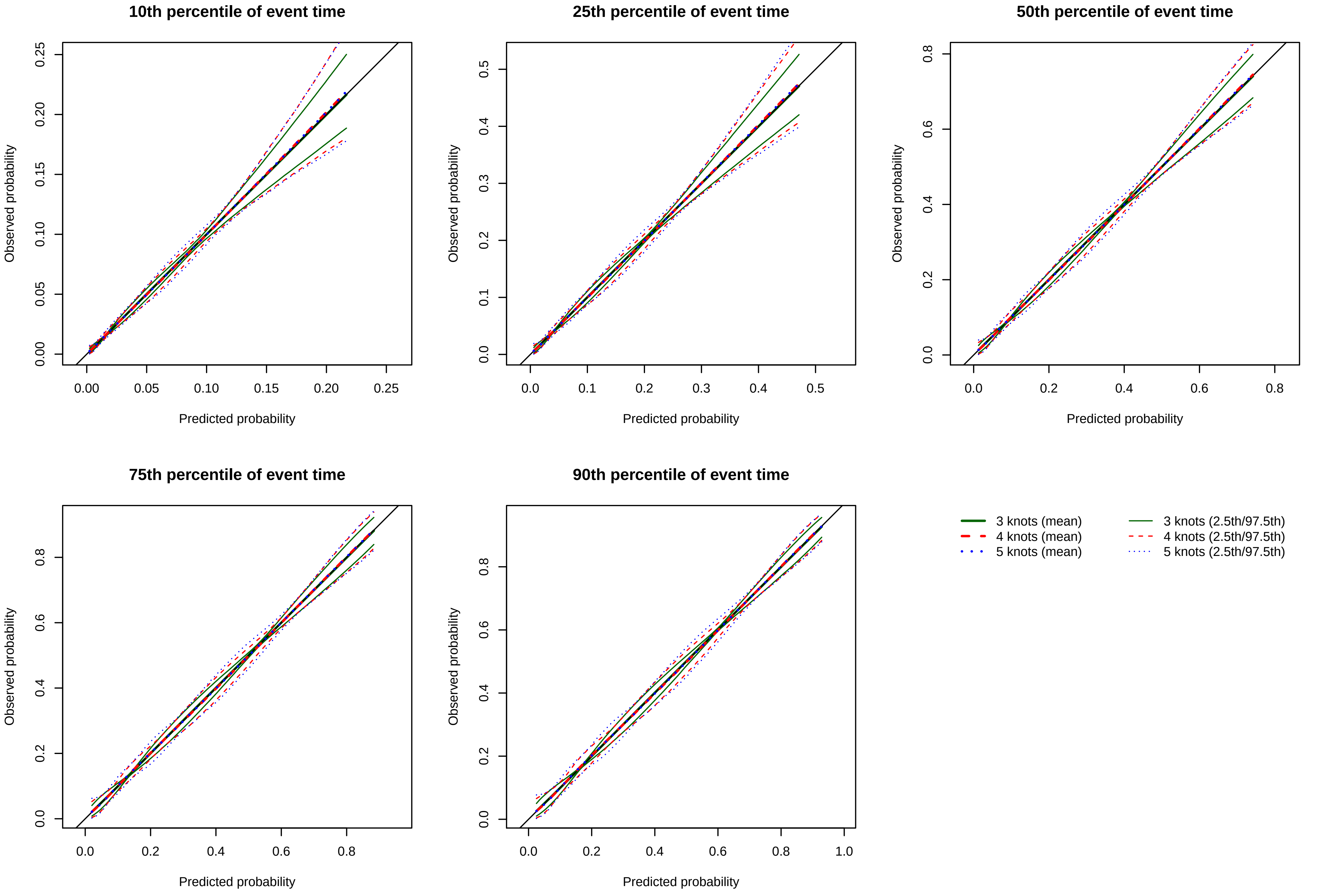

Figure A2. RCS: Choice of number of knots (p = 0.75)

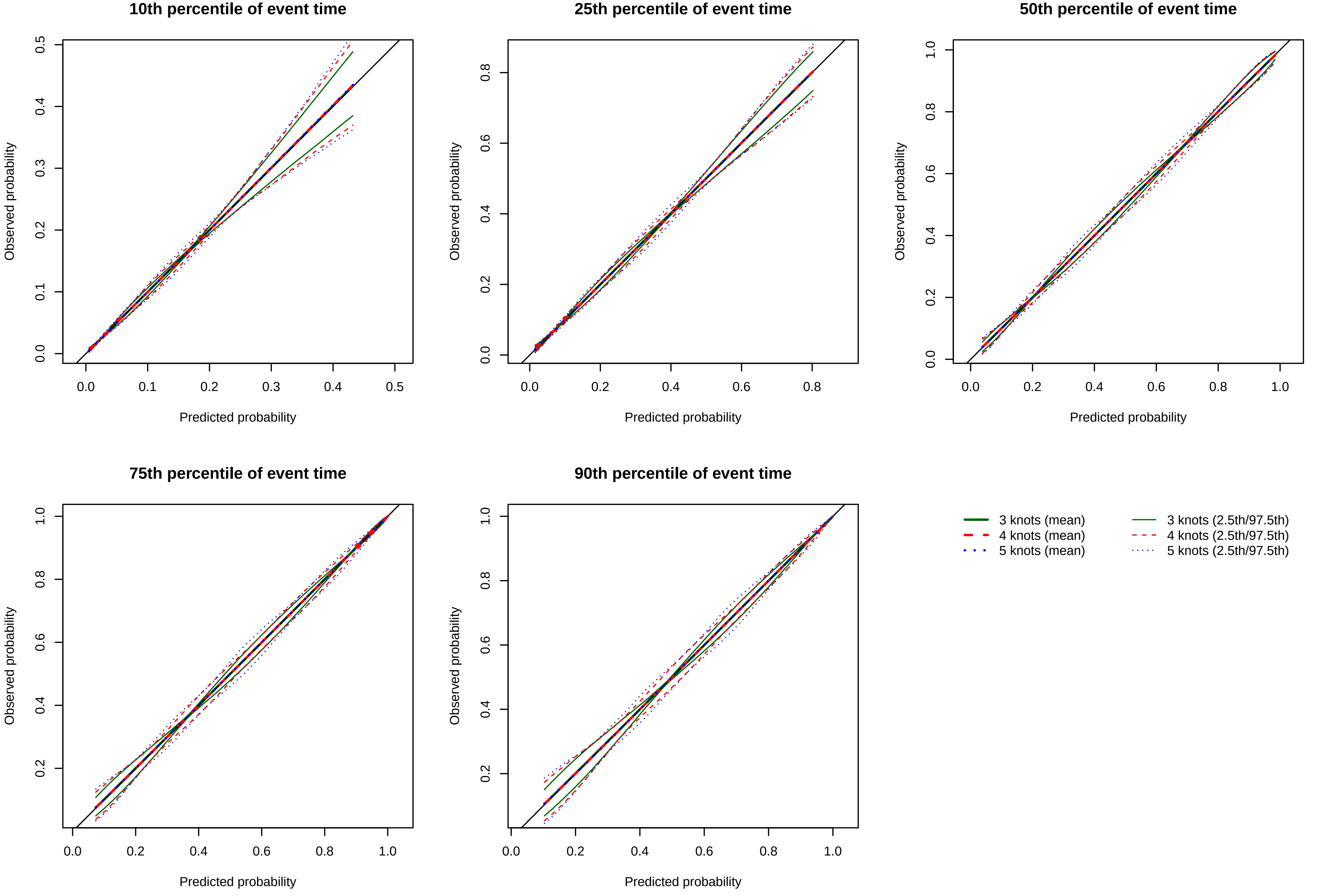

Figure A3. ICI/E50/E90 in simulations for selecting the optimal number of knots (p = 0.25)

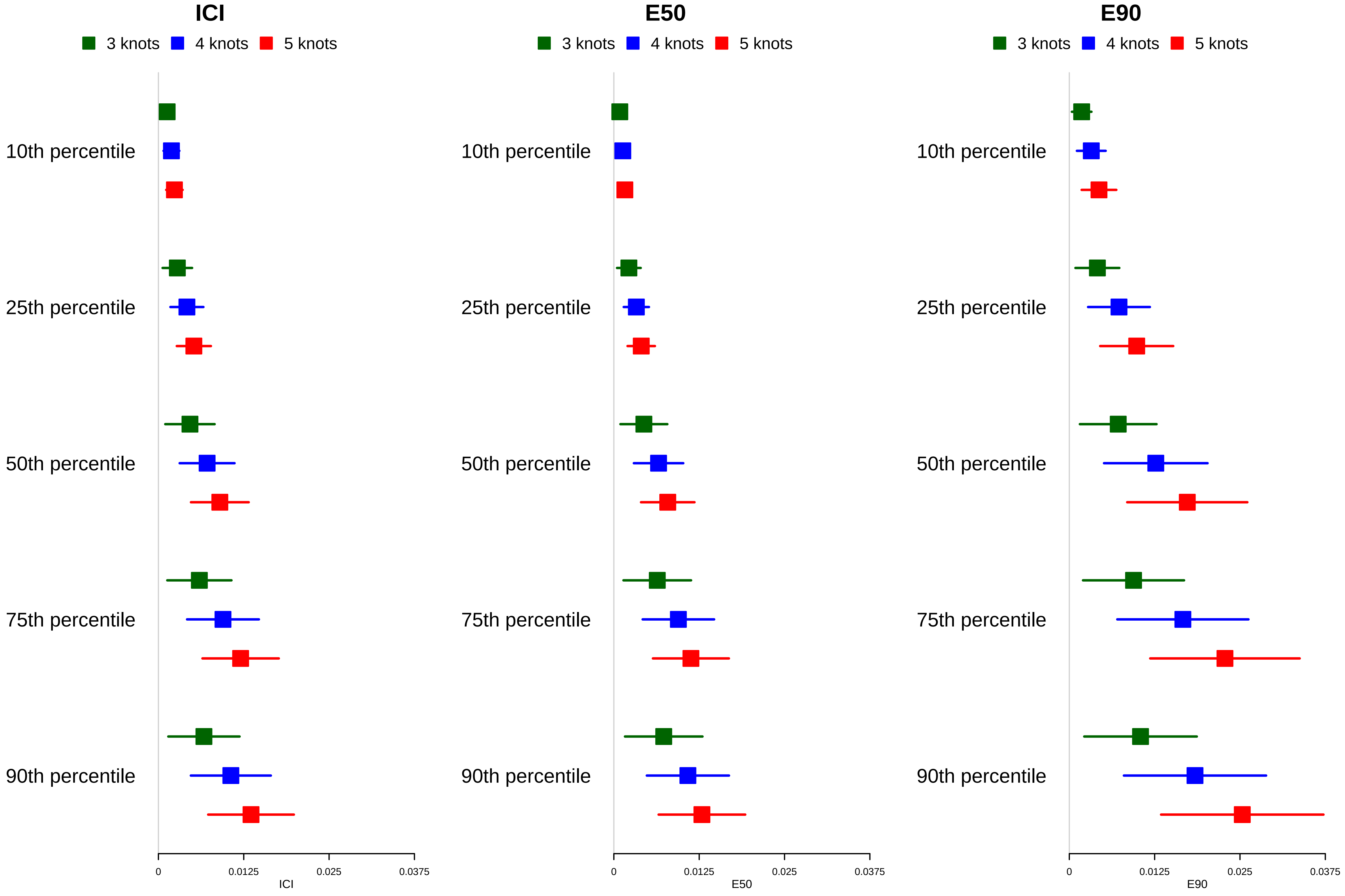

Figure A4. ICI/E50/E90 in simulations for selecting the optimal number of knots (p = 0.75)

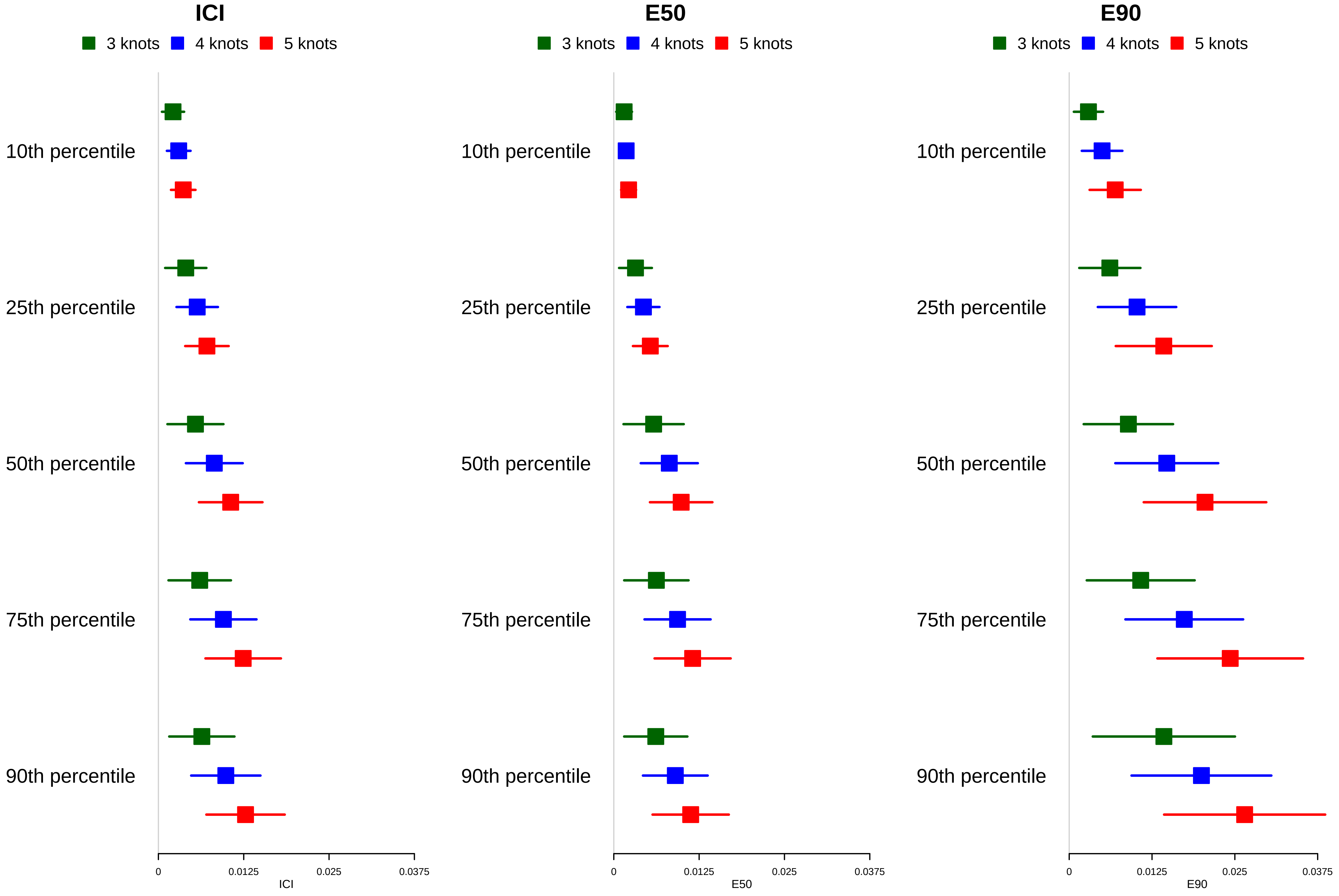

Figure A5. Effect of degree of censoring on estimated calibration curves (N = 2000 and p = 0.25)

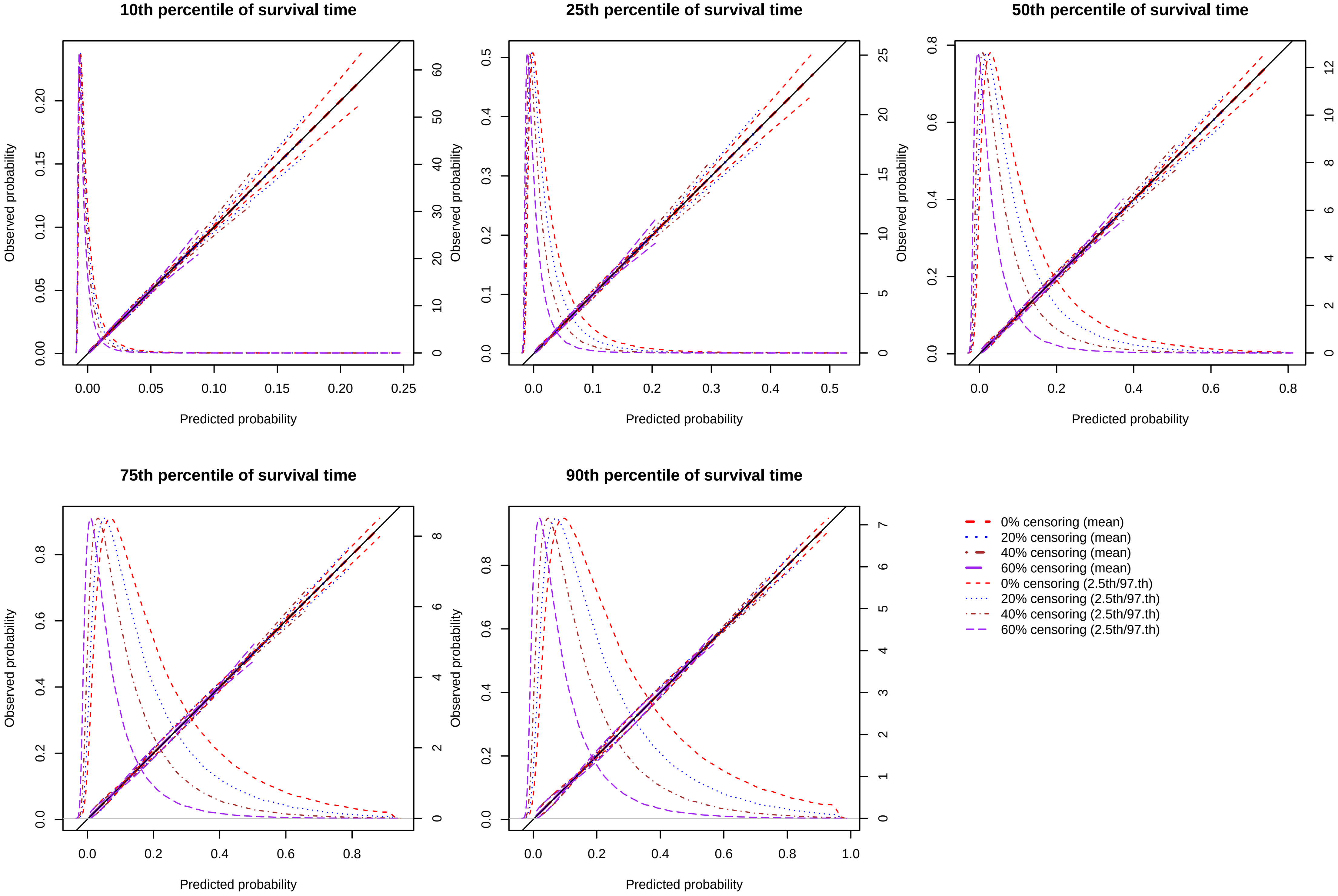

Figure A6. Effect of degree of censoring on estimated calibration curves (N = 2000 and p = 0.75)

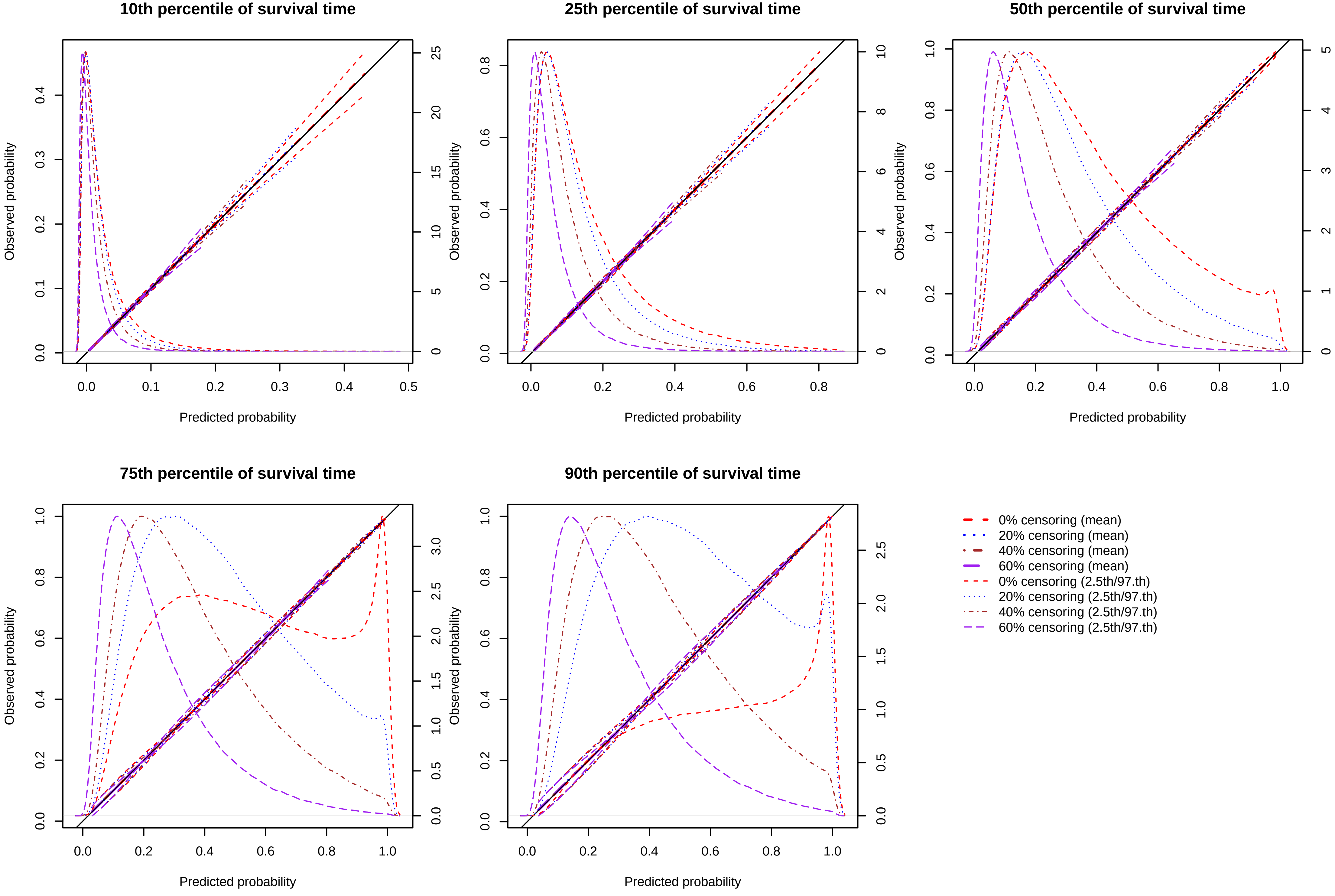

Figure A7. ICI/E90/E90 for correctly−specified model and censoring (N = 2000 and p = 0.25)

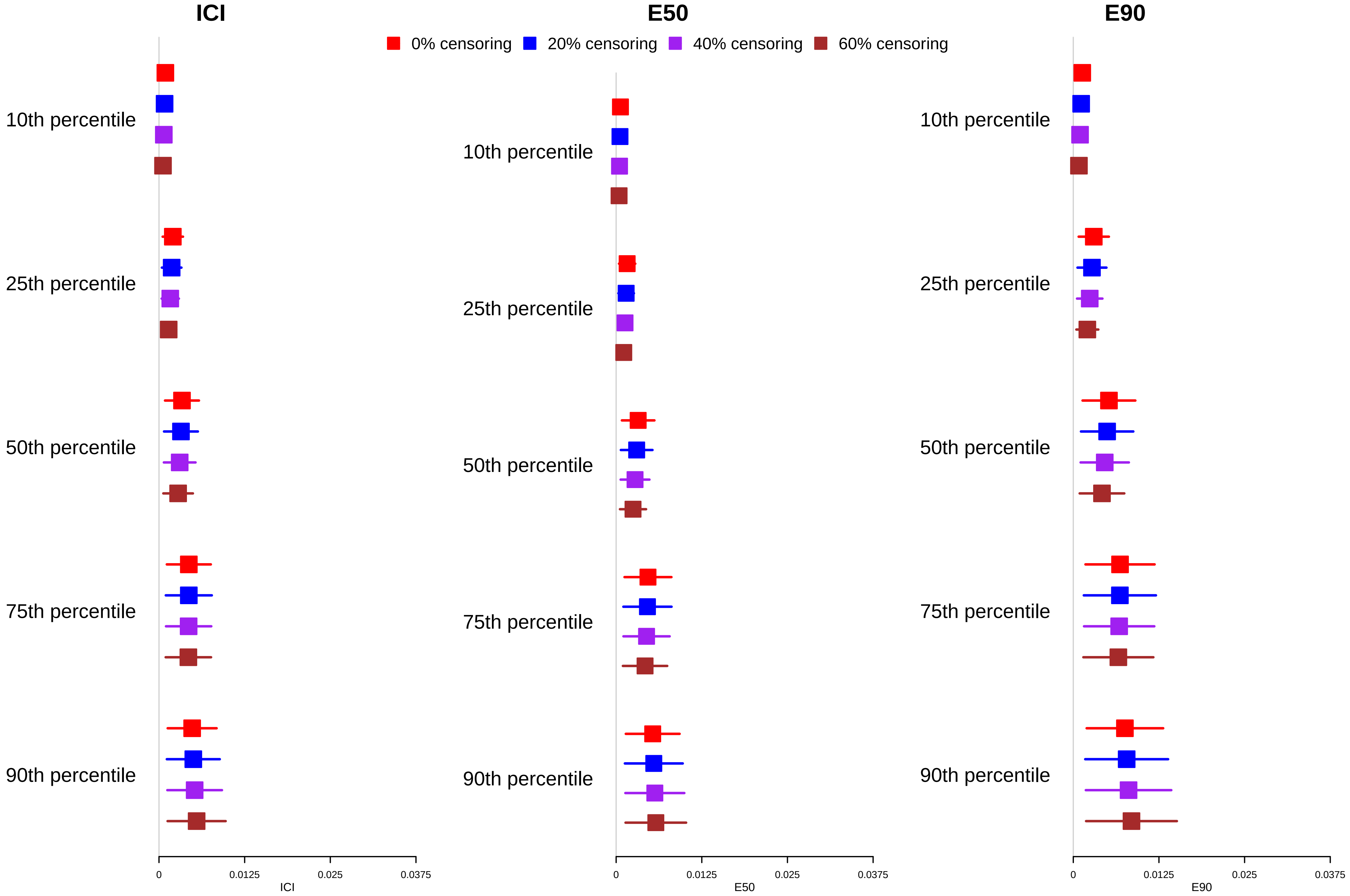

Figure A8. ICI/E90/E90 for correctly−specified model and censoring (N = 2000 and p = 0.75)

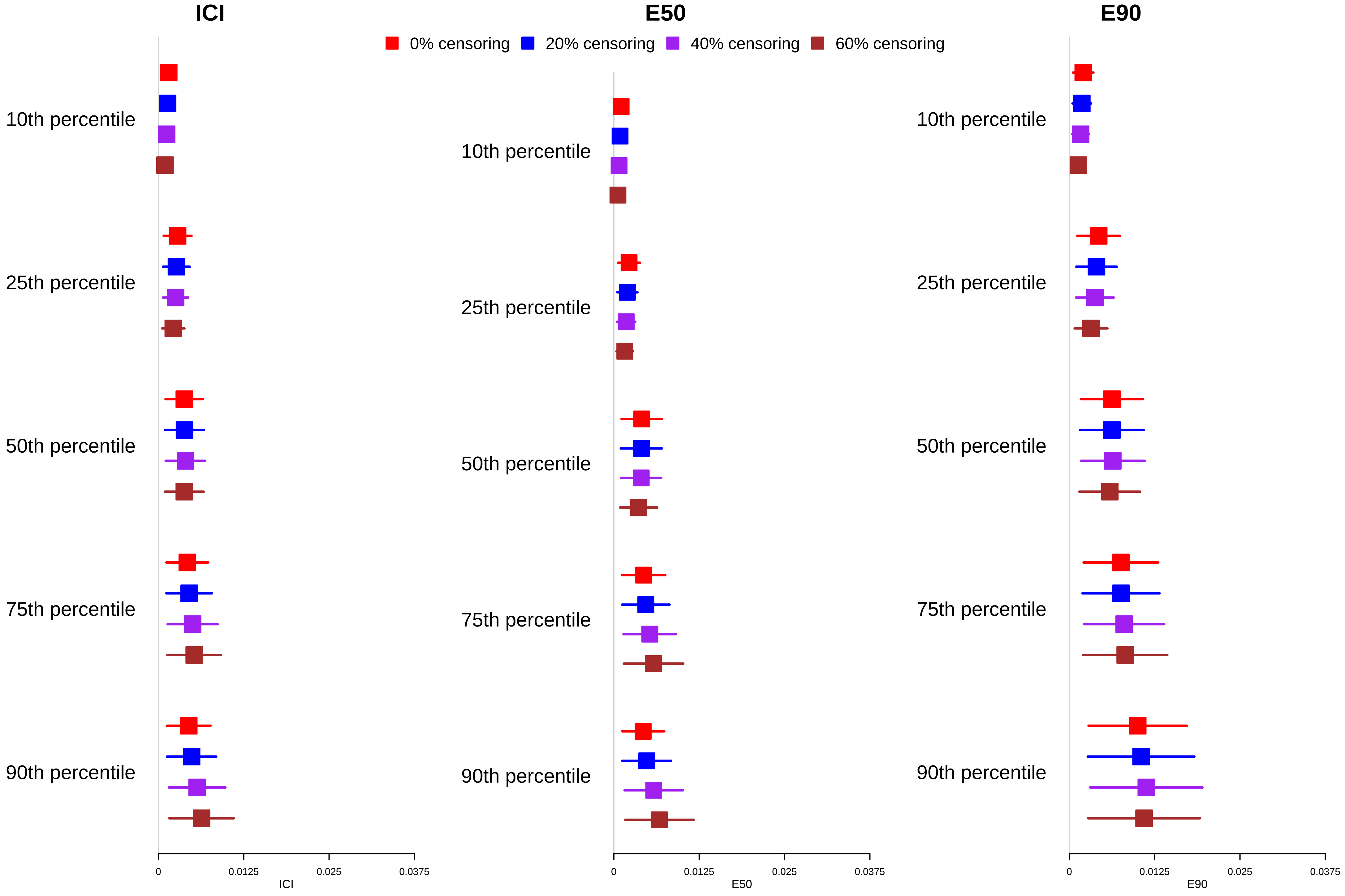

Figure A9. True model fitted with no censoring (beta1 = 0.25 & p = 0.25)

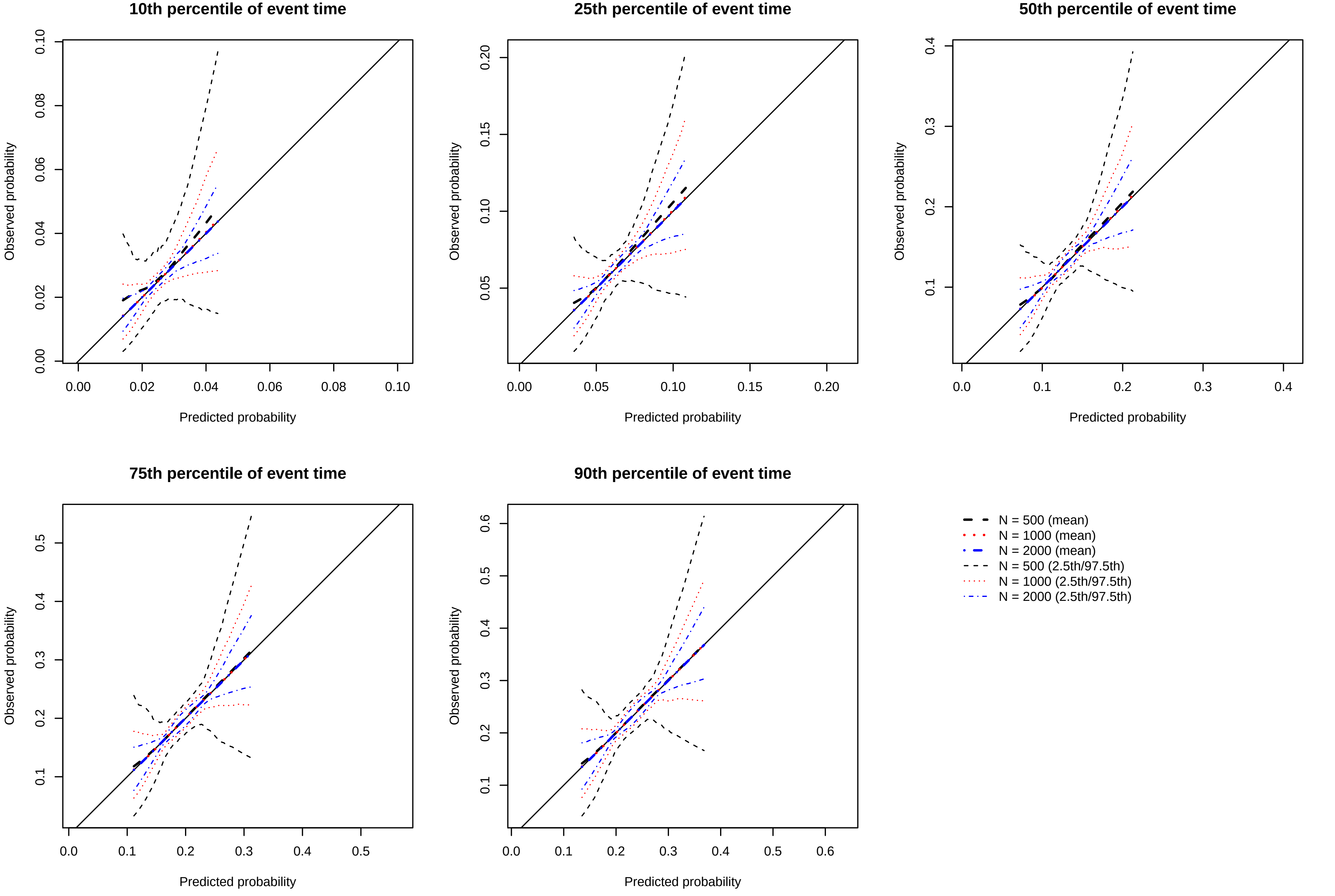

Figure A10. True model fitted with no censoring (beta1 = 0.25 & p = 0.50)

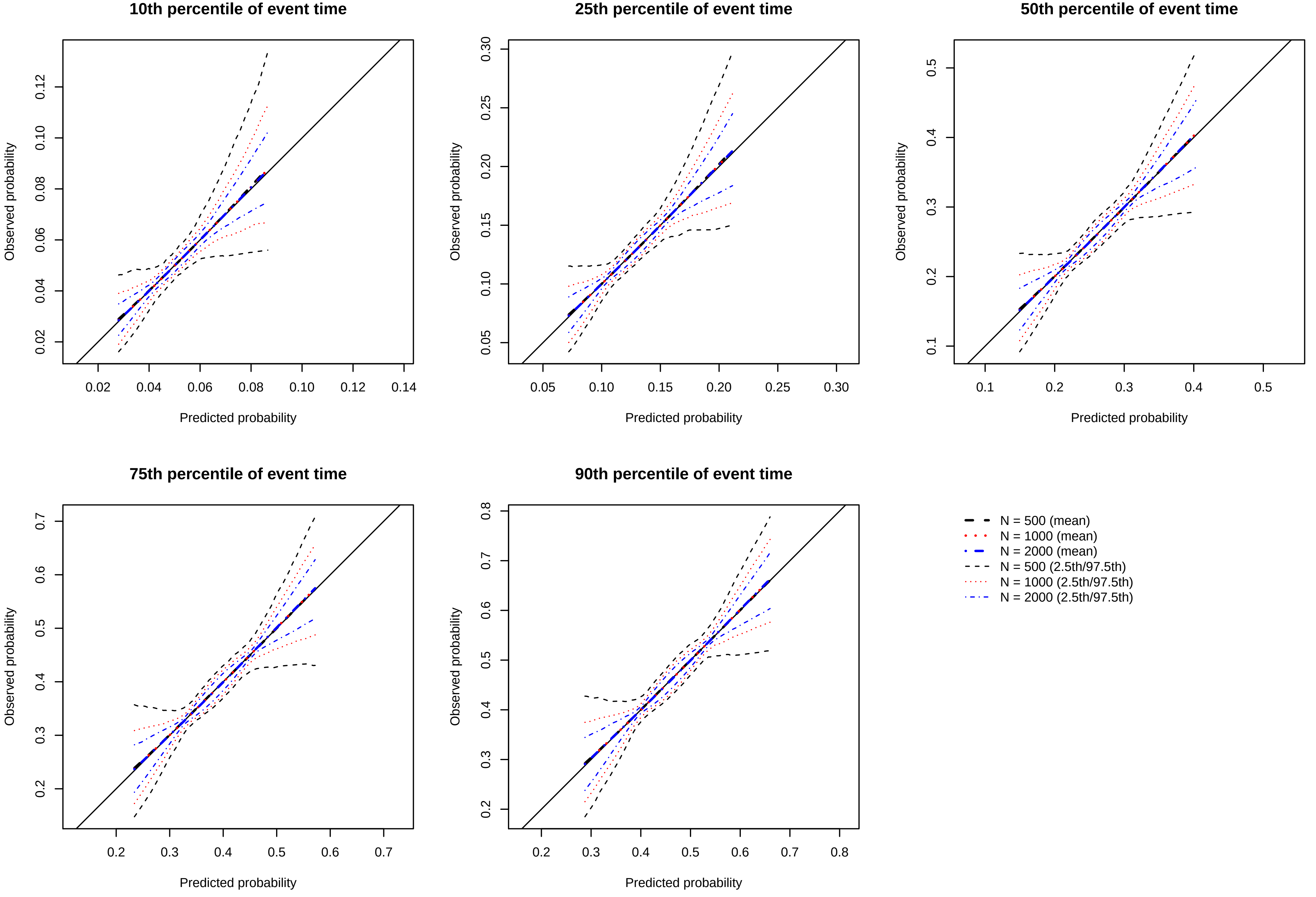

Figure A11. True model fitted with no censoring (beta1 = 0.25 & p = 0.75)

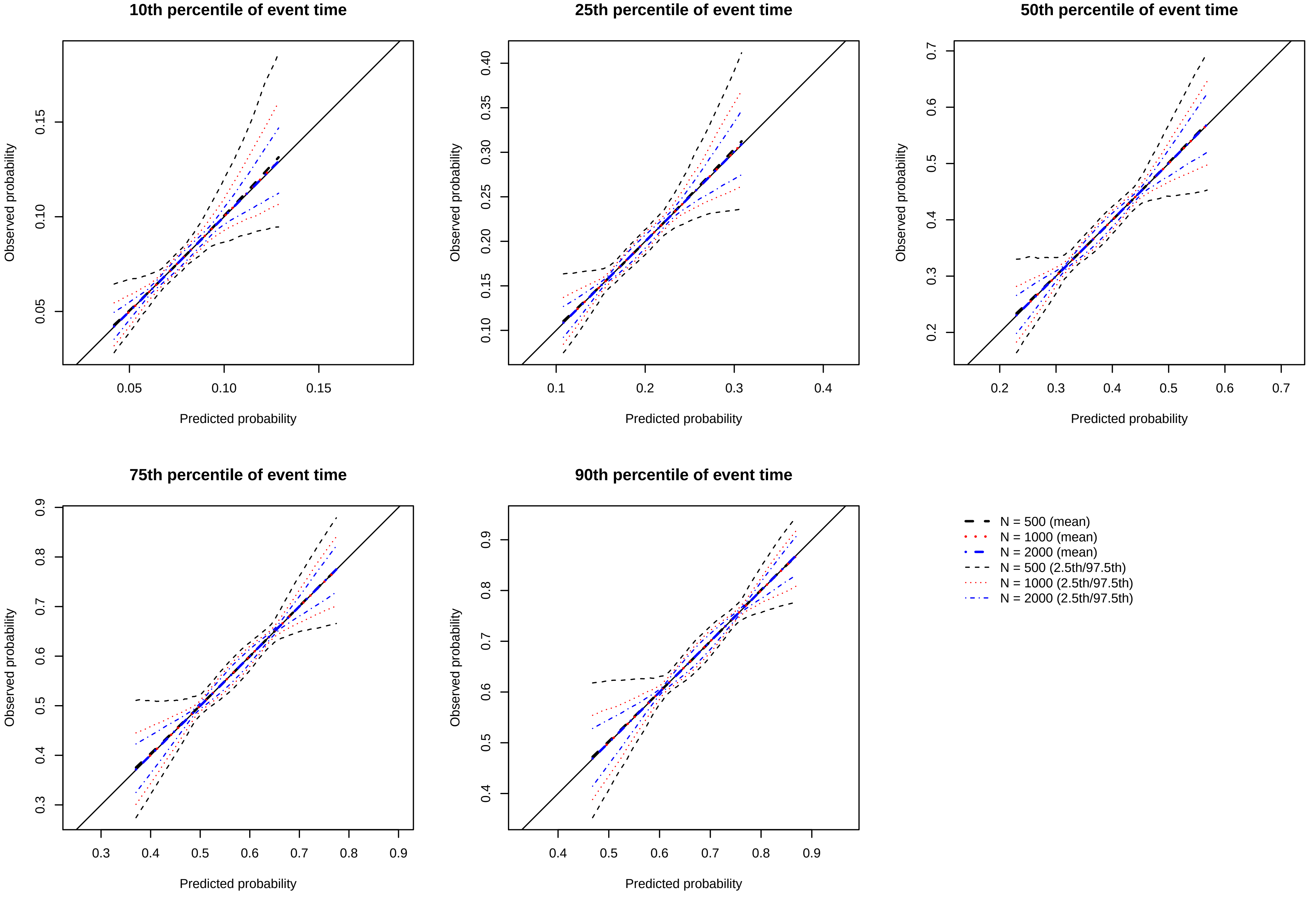

Figure A12. True model fitted with no censoring (beta1 = 0.50 & p = 0.25)

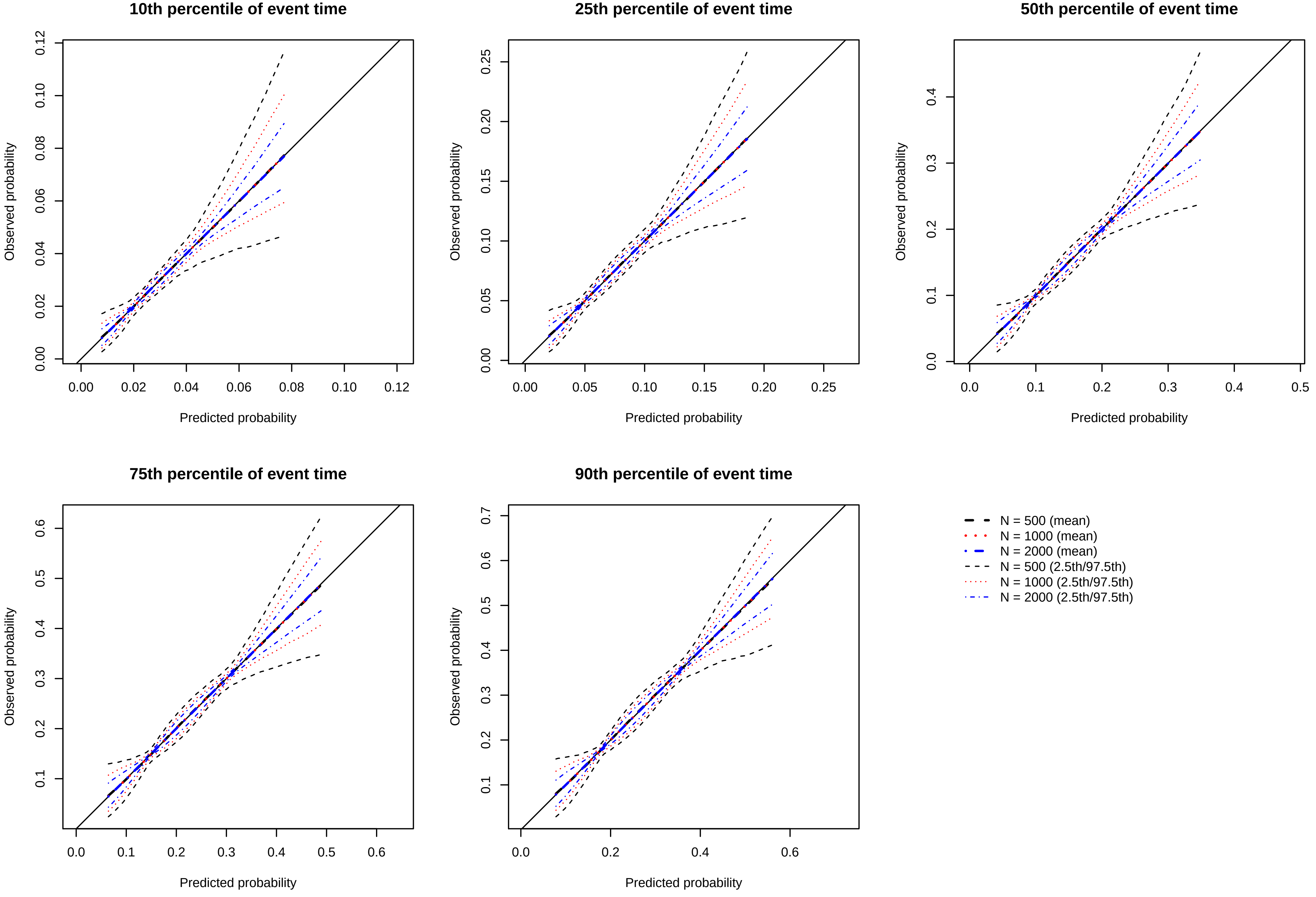

Figure A13. True model fitted with no censoring (beta1 = 0.50 & p = 0.75)

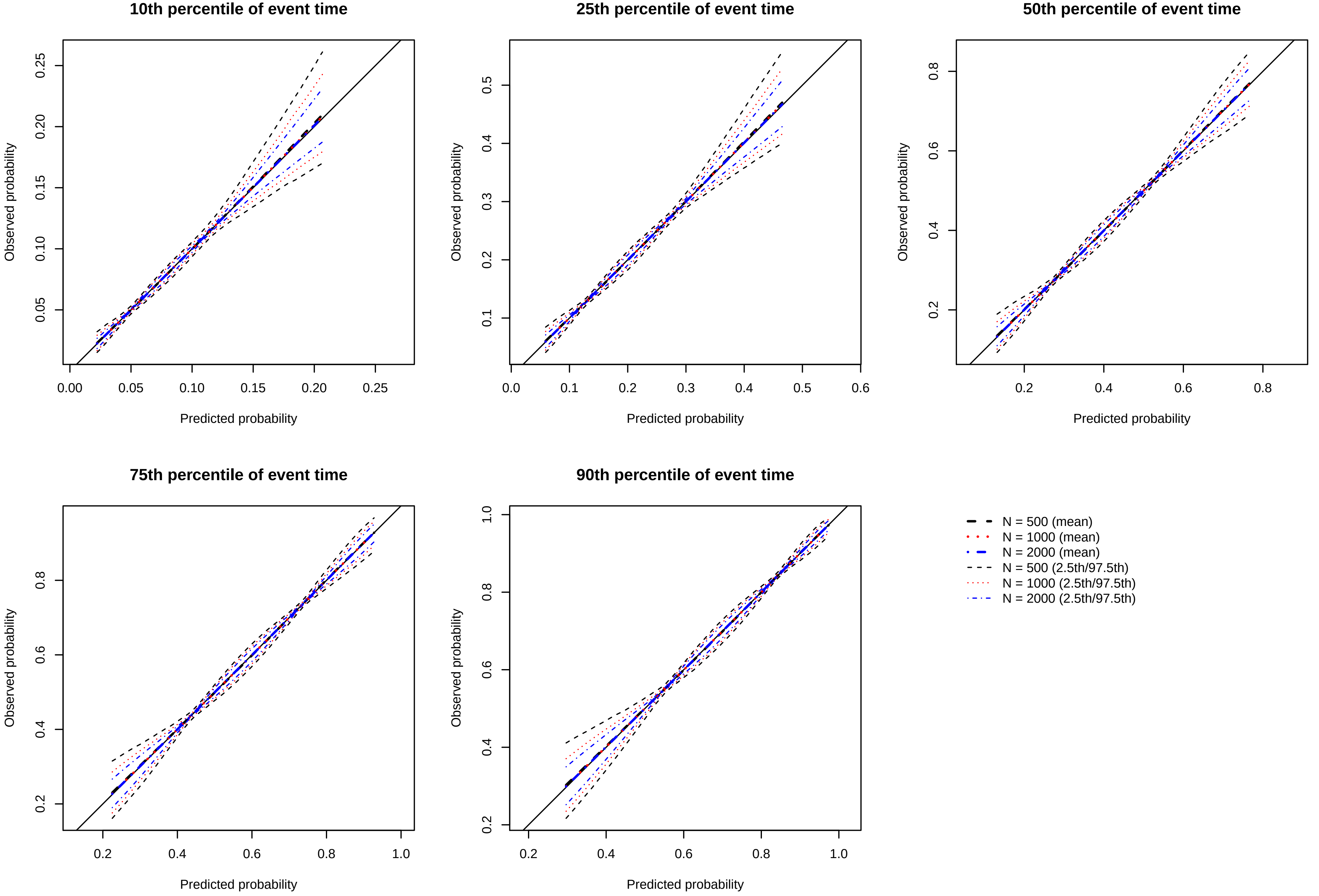

Figure A14. True model fitted with no censoring (beta1 = 1 & p = 0.25)

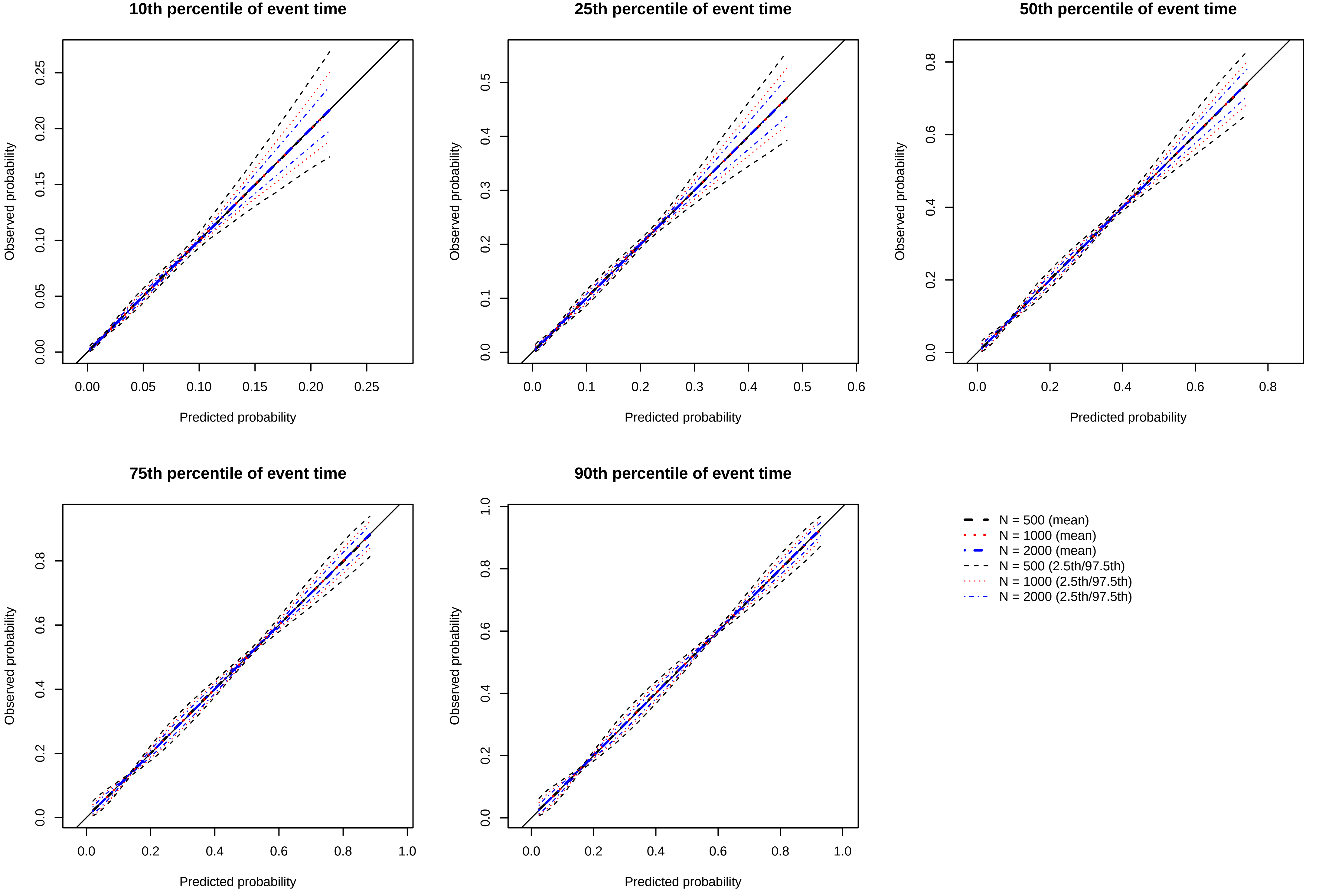

Figure A15. True model fitted with no censoring (beta1 = 1 & p = 0.50)

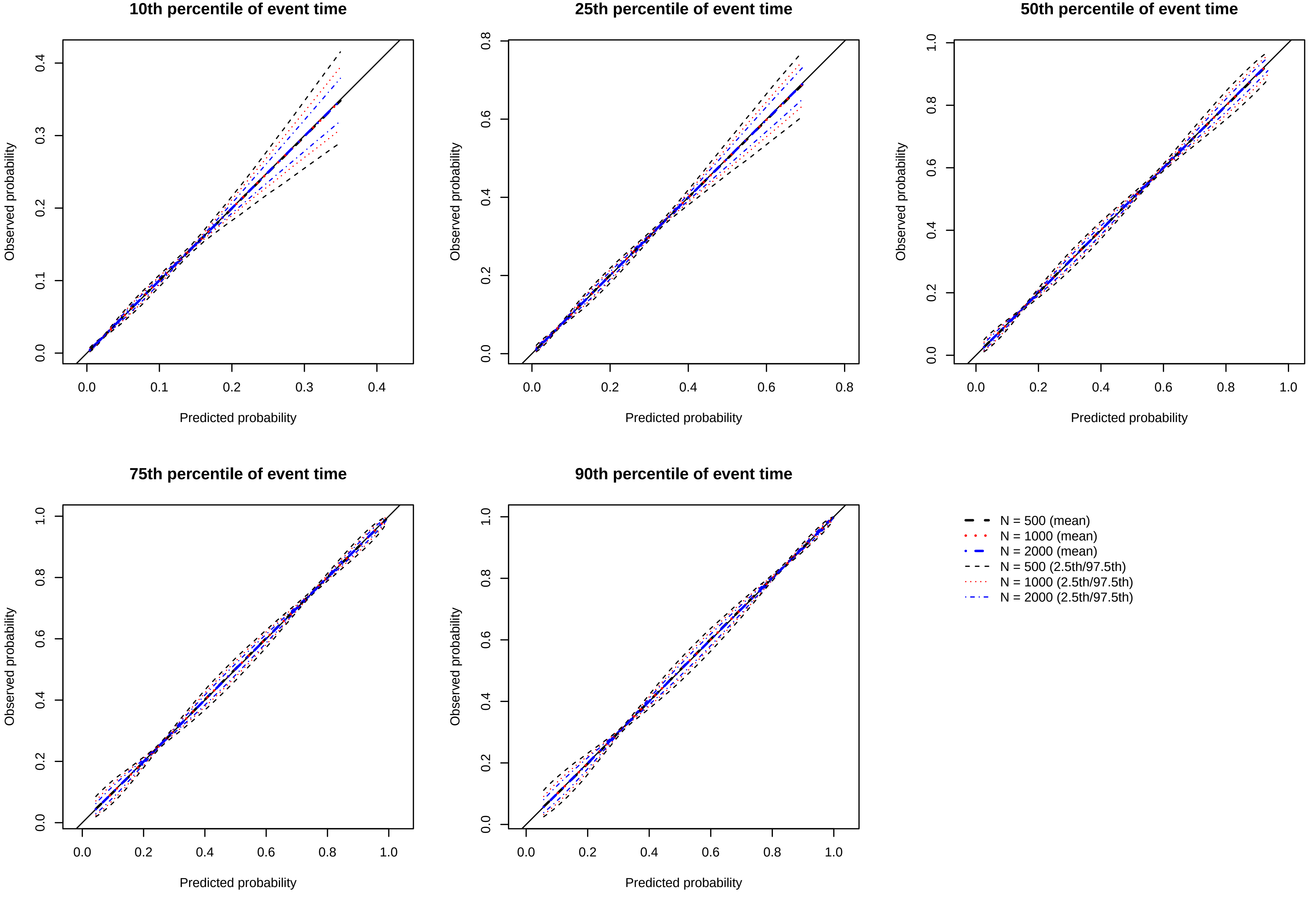

Figure A16. True model fitted with no censoring (beta1 = 1 & p = 0.75)

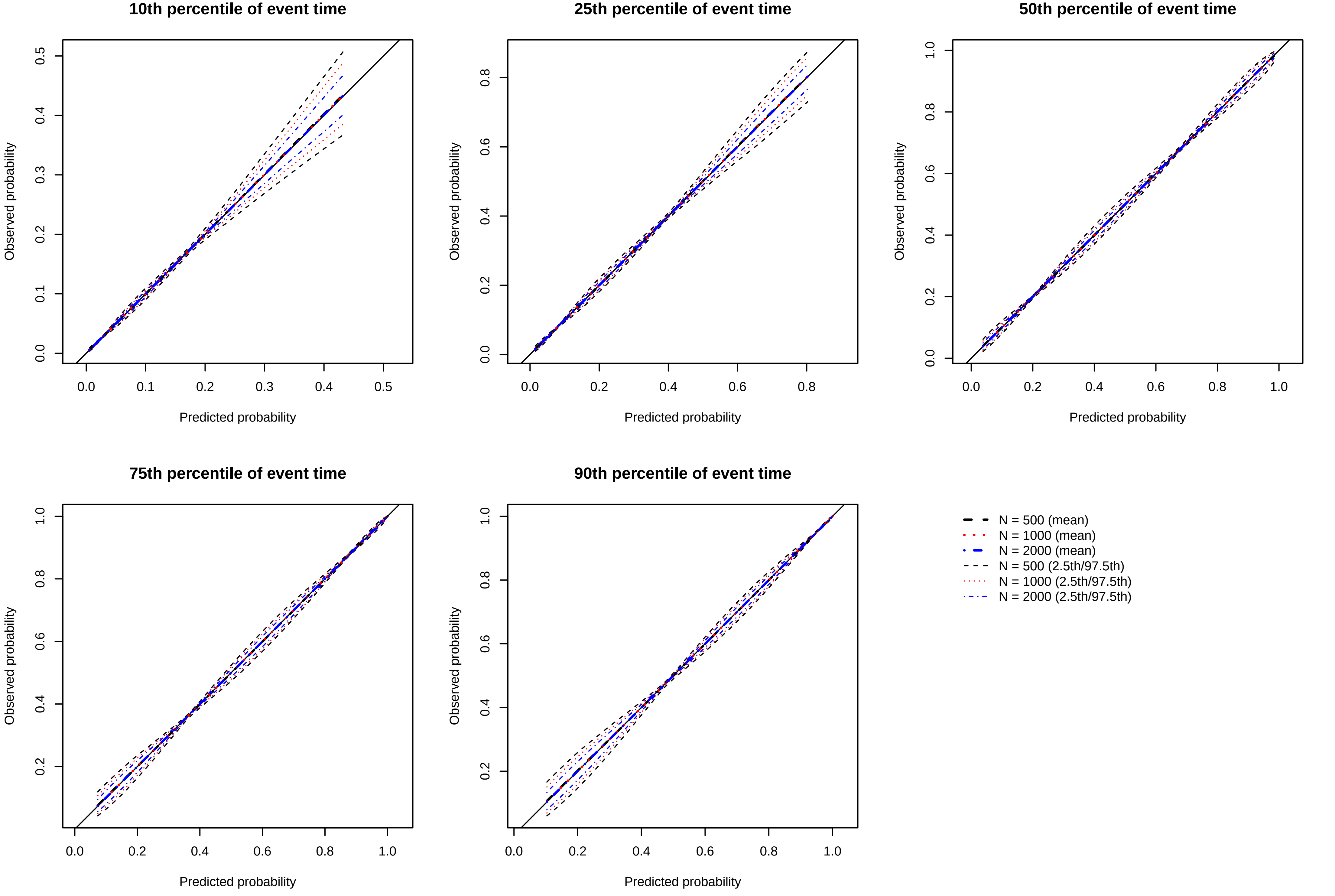

Figure A17. ICI/E90/E90 for correctly−specified model without censoring (p = 0.25)

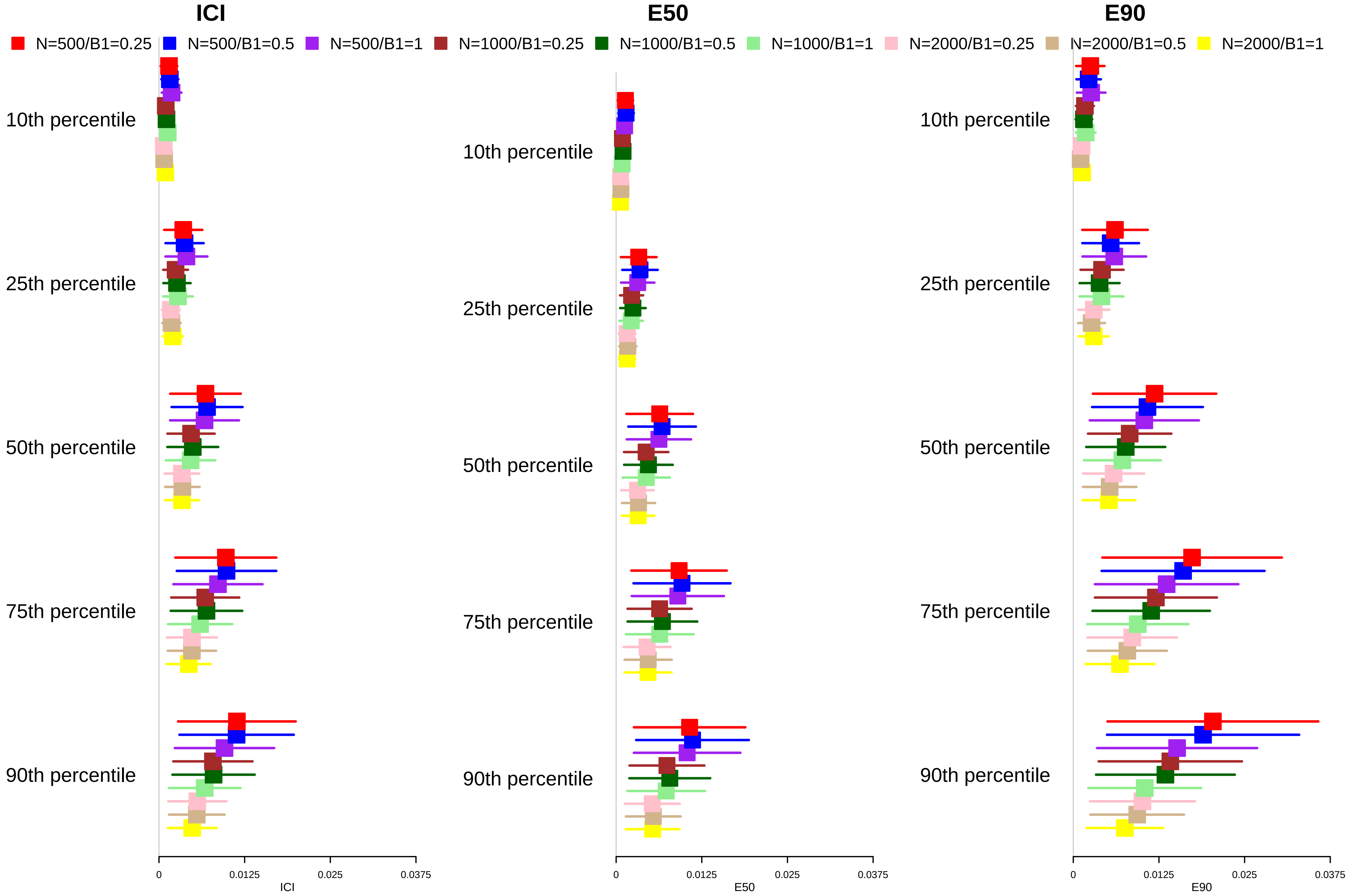

Figure A18. ICI/E90/E90 for correctly−specified model without censoring (p = 0.75)

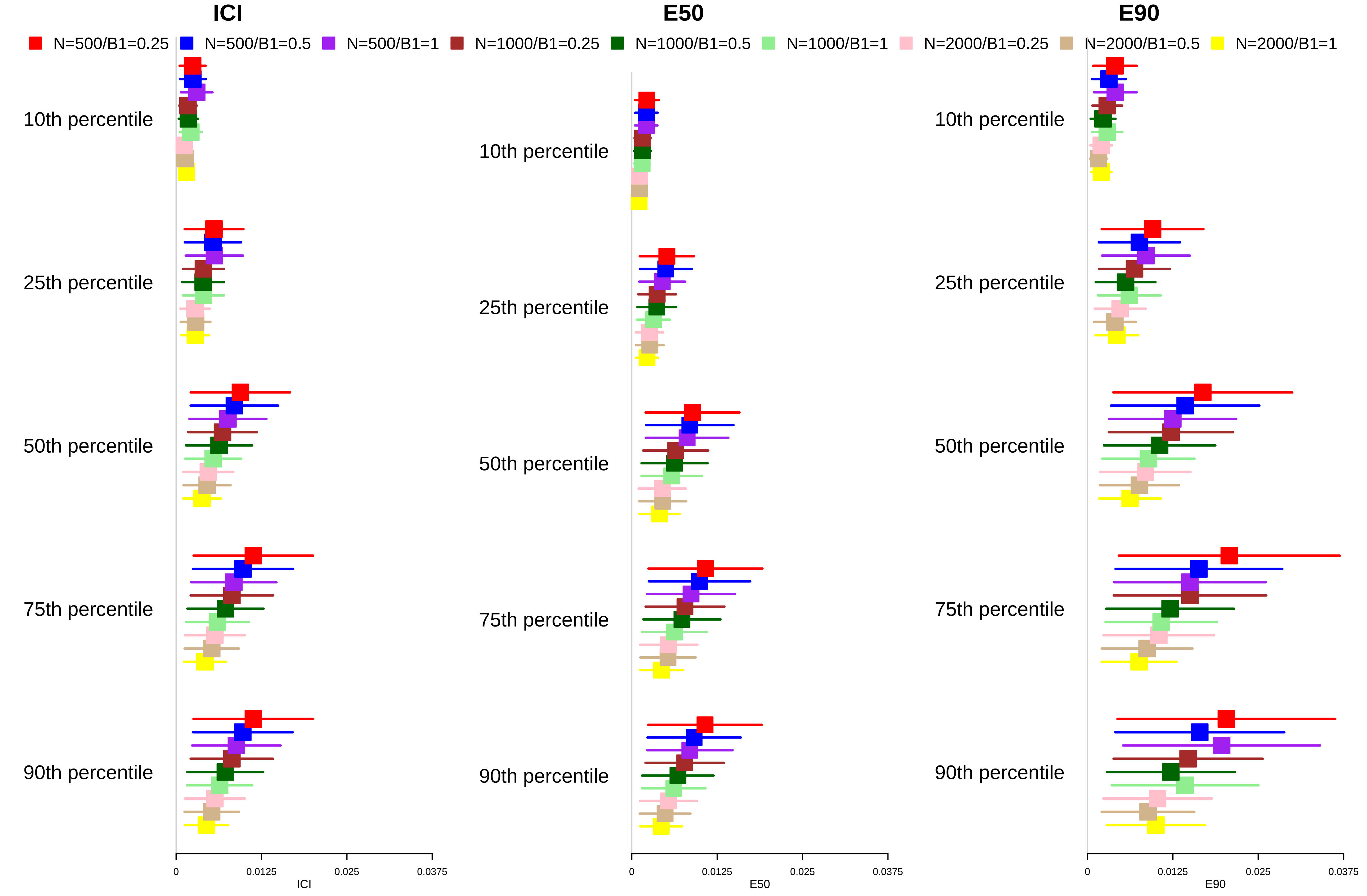

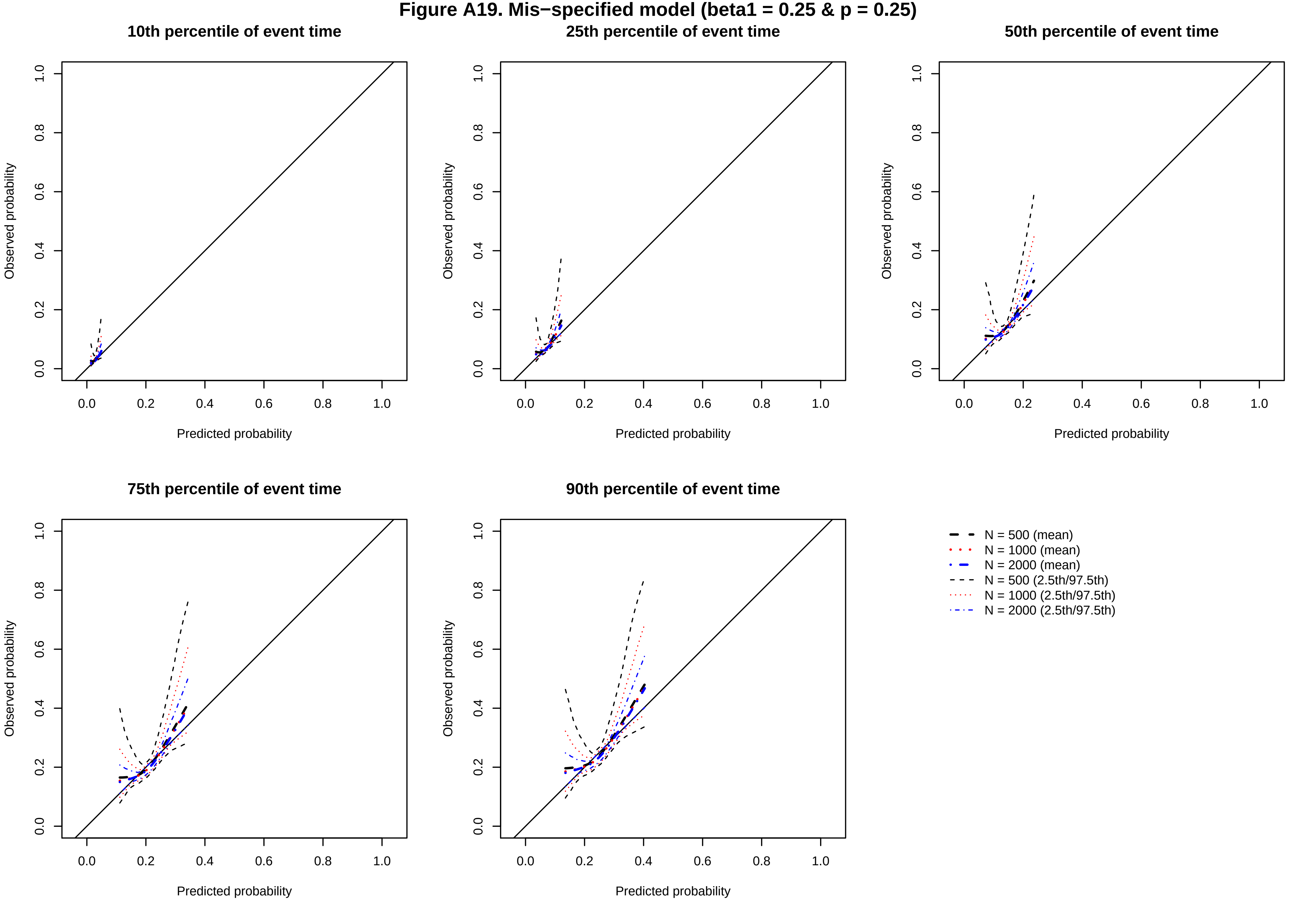
Figure A19. Mis−specified model (beta1 = 0.25 & p = 0.25)
10th percentile of event time
25th percentile of event time
50th percentile of event time
75th percentile of event time
90th percentile of event time
Observed probability
Predicted probability
0.0
0.2
0.4
0.6
0.8
1.0
N = 500 (mean)
N = 1000 (mean)
N = 2000 (mean)
N = 500 (2.5th/97.5th)
N = 1000 (2.5th/97.5th)
N = 2000 (2.5th/97.5th)

Figure A20. Mis−specified model (beta1 = 0.25 & p = 0.50)

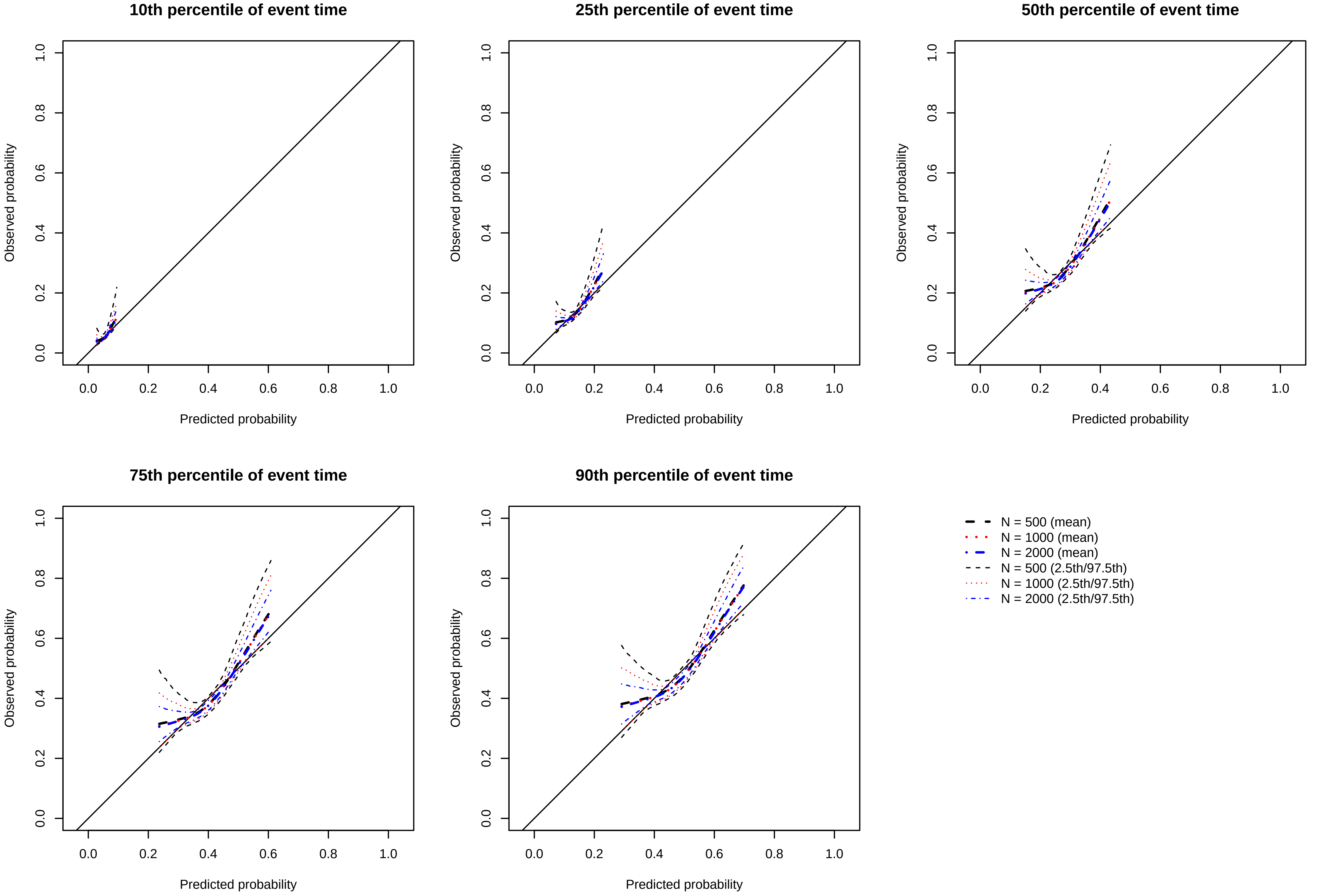

Figure A21. Mis−specified model (beta1 = 0.25 & p = 0.75)

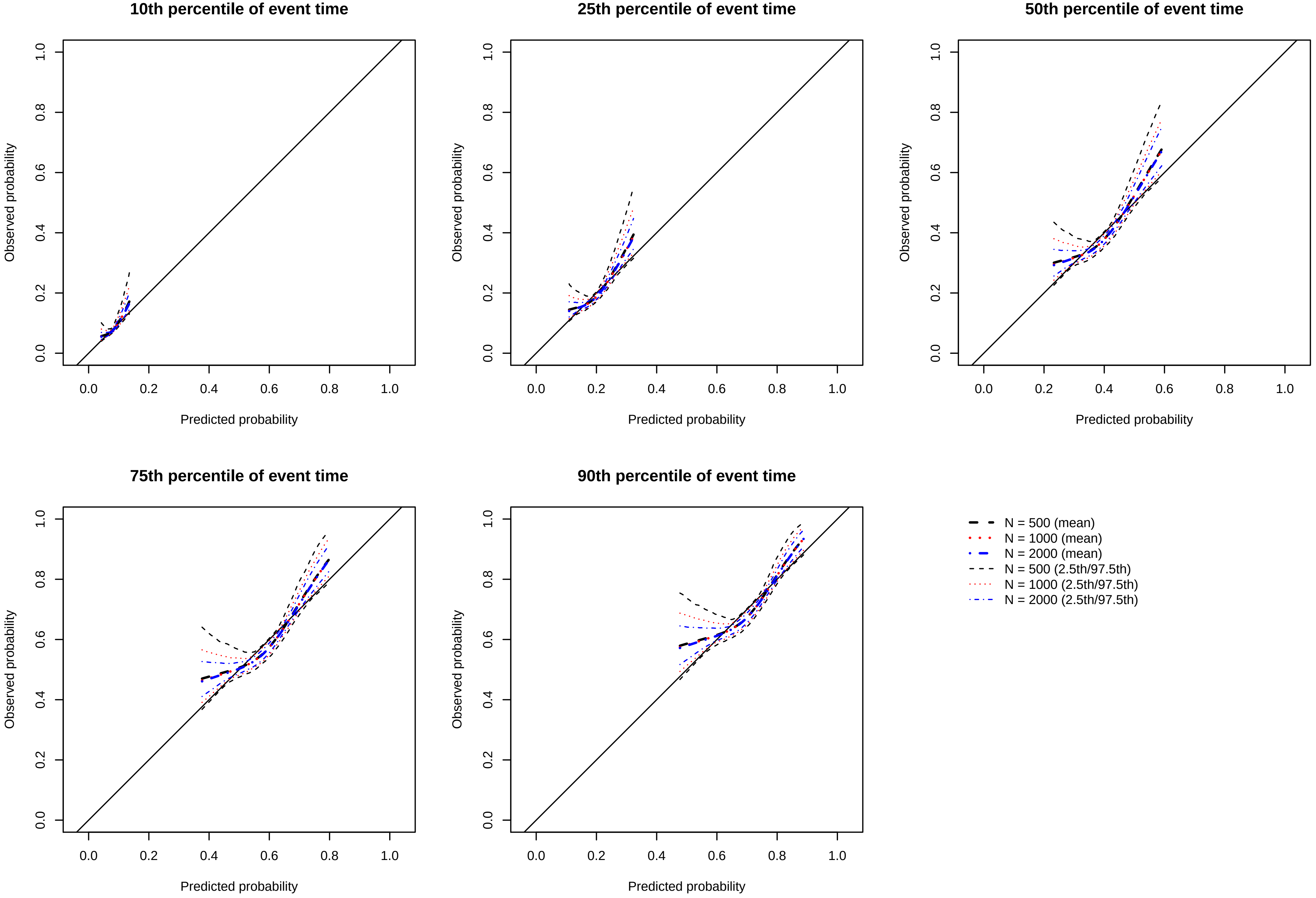

Figure A22. Mis−specified model (beta1 = 0.50 & p = 0.25)

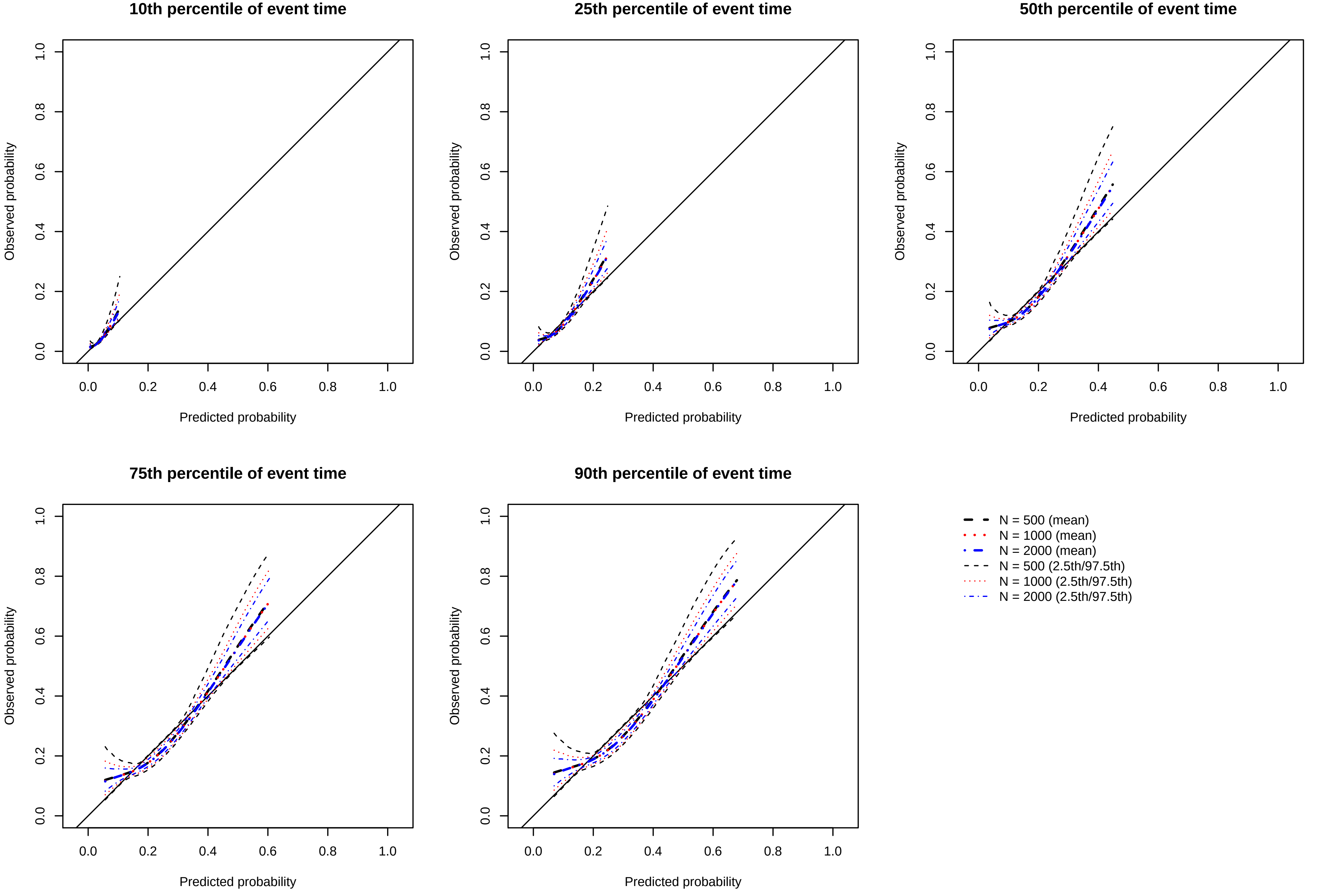

Figure A23. Mis−specified model (beta1 = 0.50 & p = 0.75)

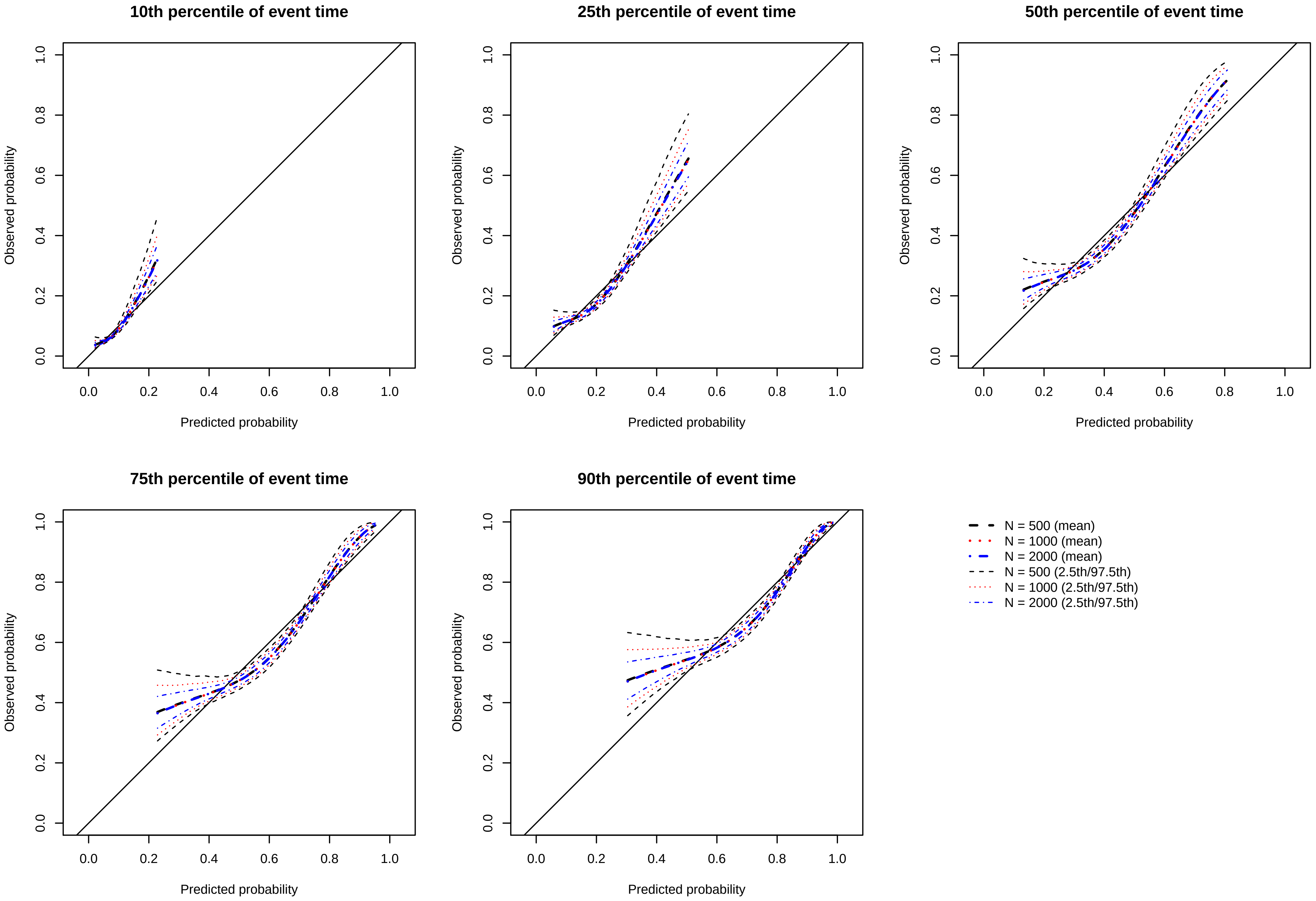

Figure A24. Mis−specified model (beta1 = 1 & p = 0.25)

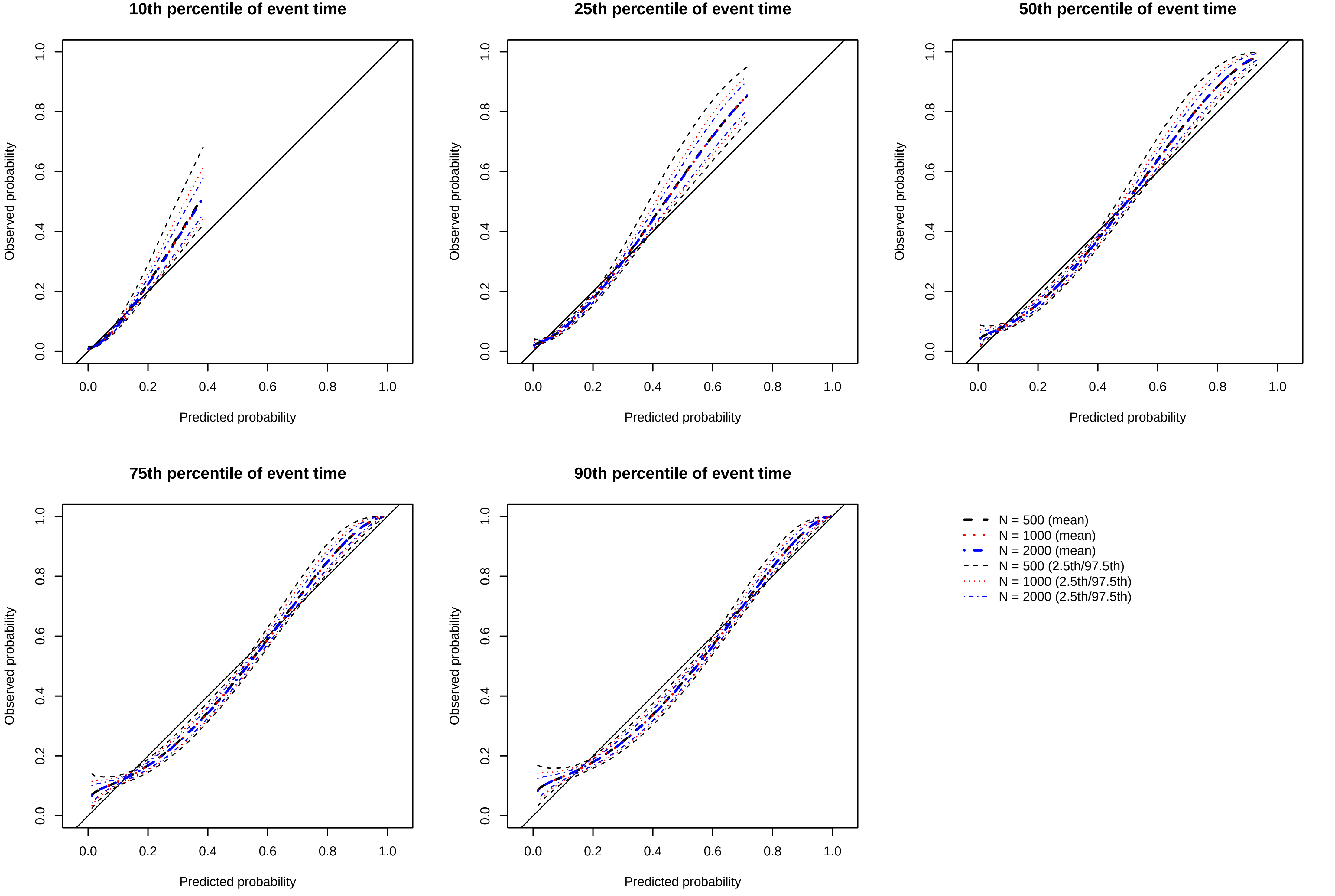

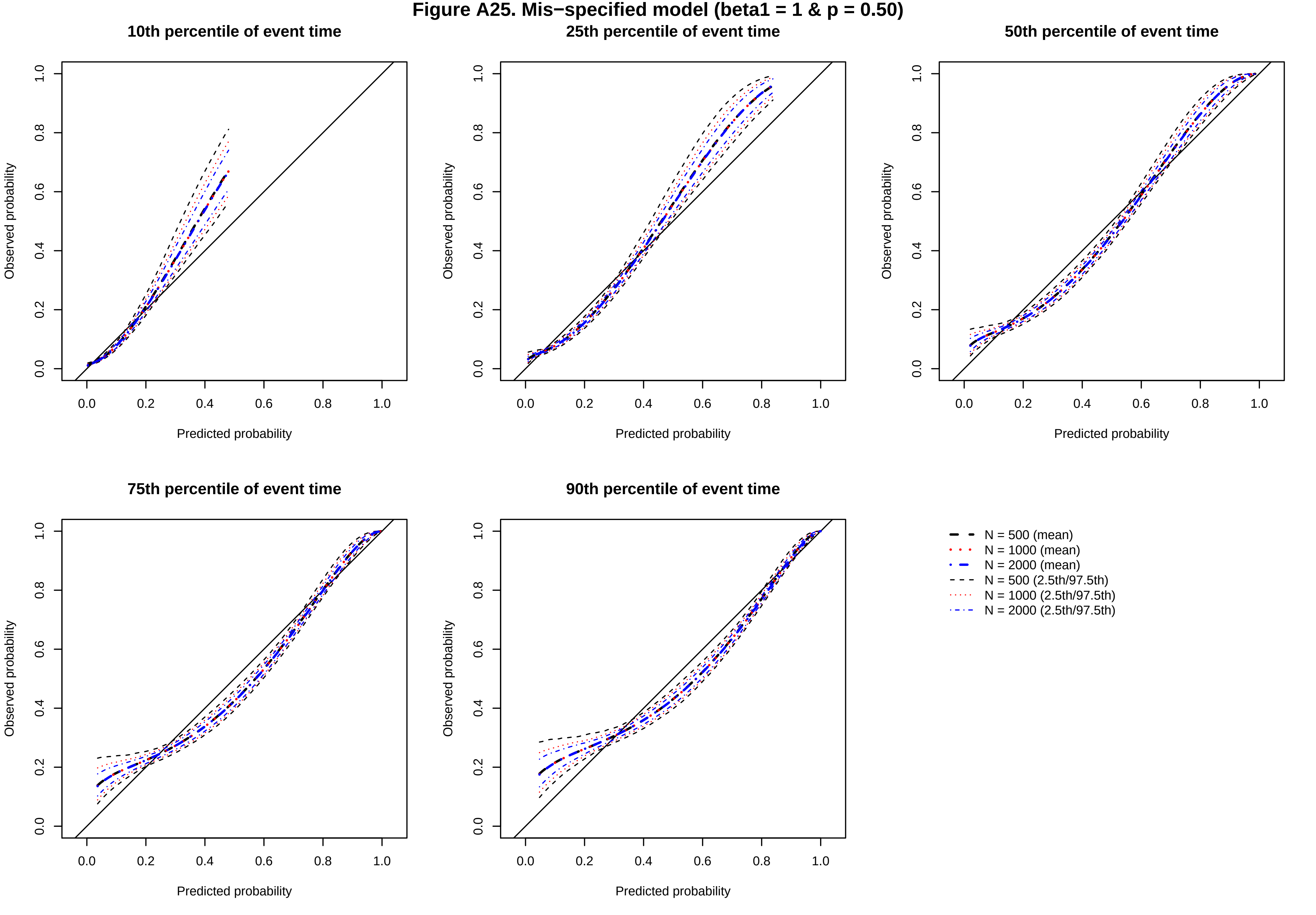
Figure A25. Mis−specified model (beta1 = 1 & p = 0.50)
10th percentile of event time
25th percentile of event time
50th percentile of event time
75th percentile of event time
90th percentile of event time
Observed probability
Predicted probability
0.0
0.2
0.4
0.6
0.8
1.0
N = 500 (mean)
N = 1000 (mean)
N = 2000 (mean)
N = 500 (2.5th/97.5th)
N = 1000 (2.5th/97.5th)
N = 2000 (2.5th/97.5th)

Figure A26. Mis−specified model (beta1 = 1 & p = 0.75)

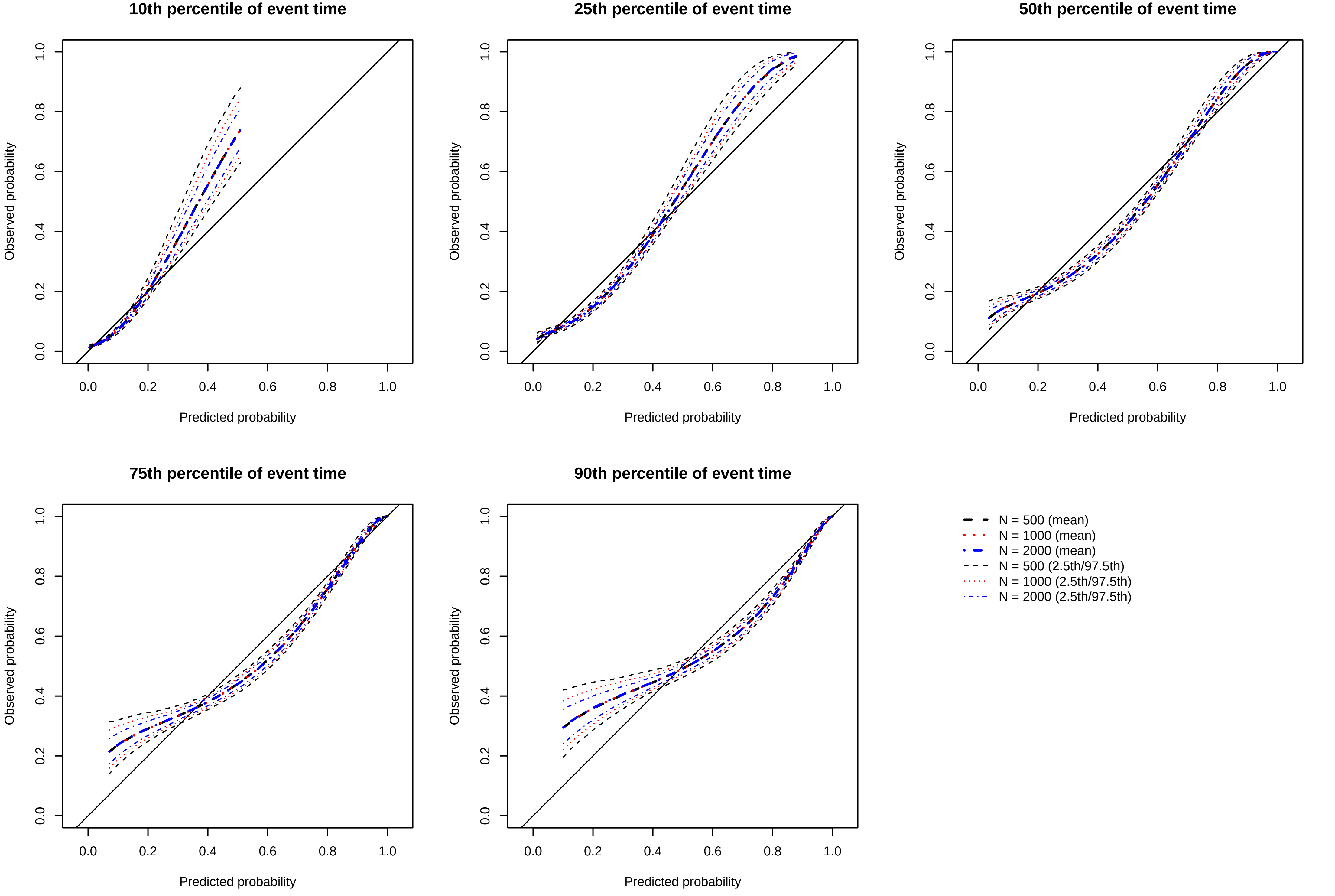

Figure A27. ICI/E90/E90 for incorrectly−specified model (p = 0.25)

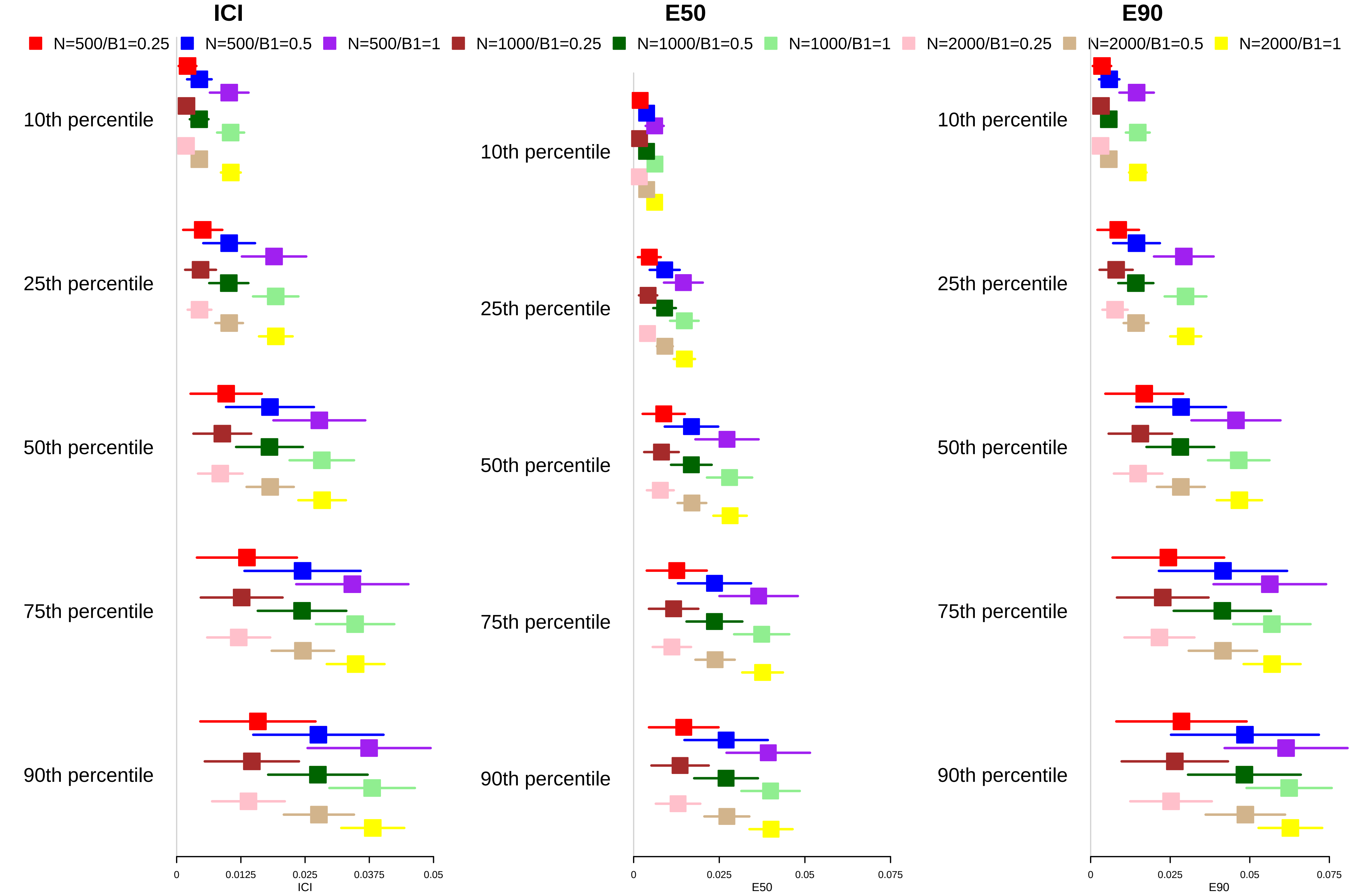

Figure A28. ICI/E90/E90 for incorrectly−specified model (p = 0.75)

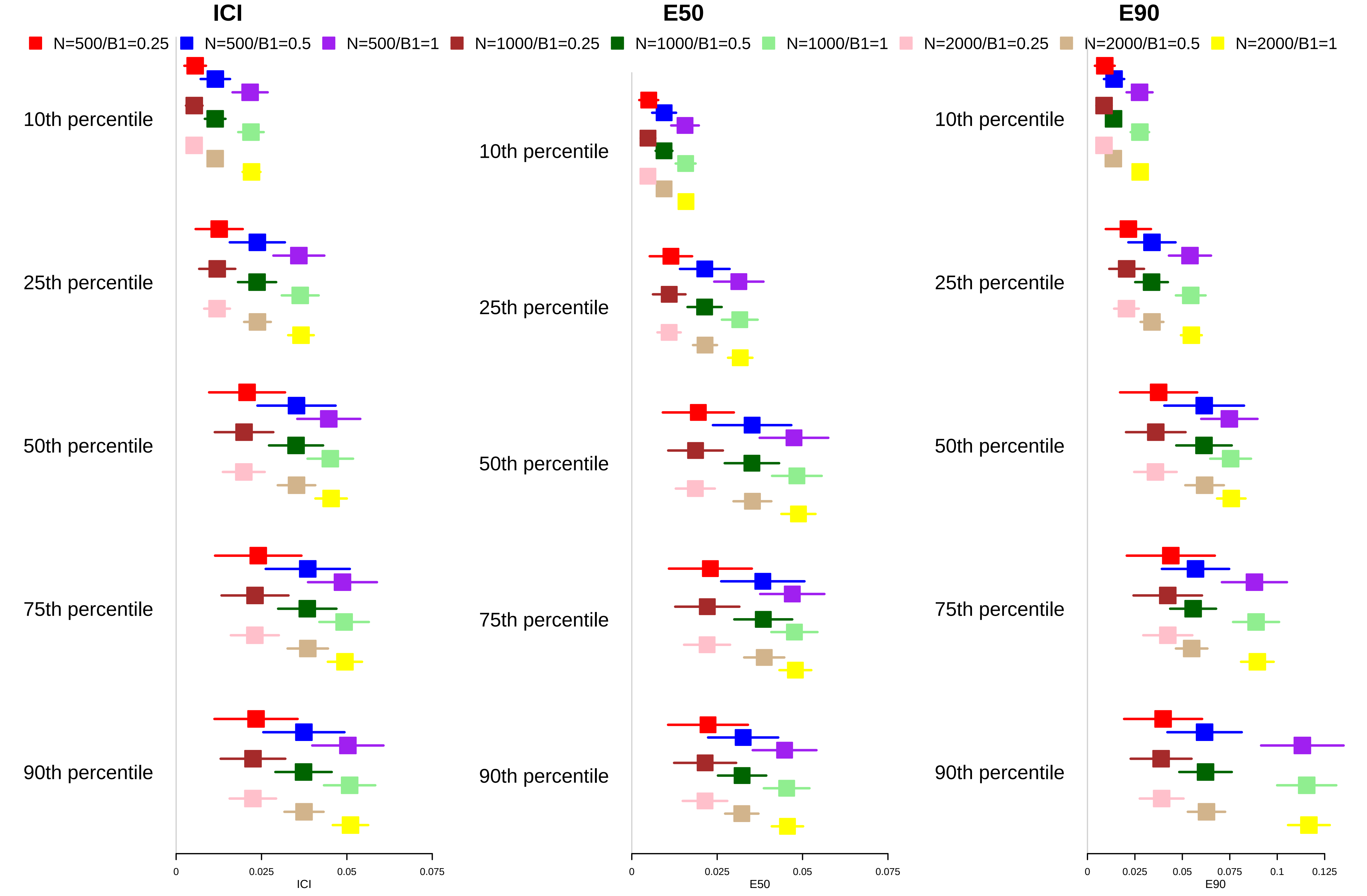

Figure A29. Mis−specified model (omission of main effect) (rho=0.25)

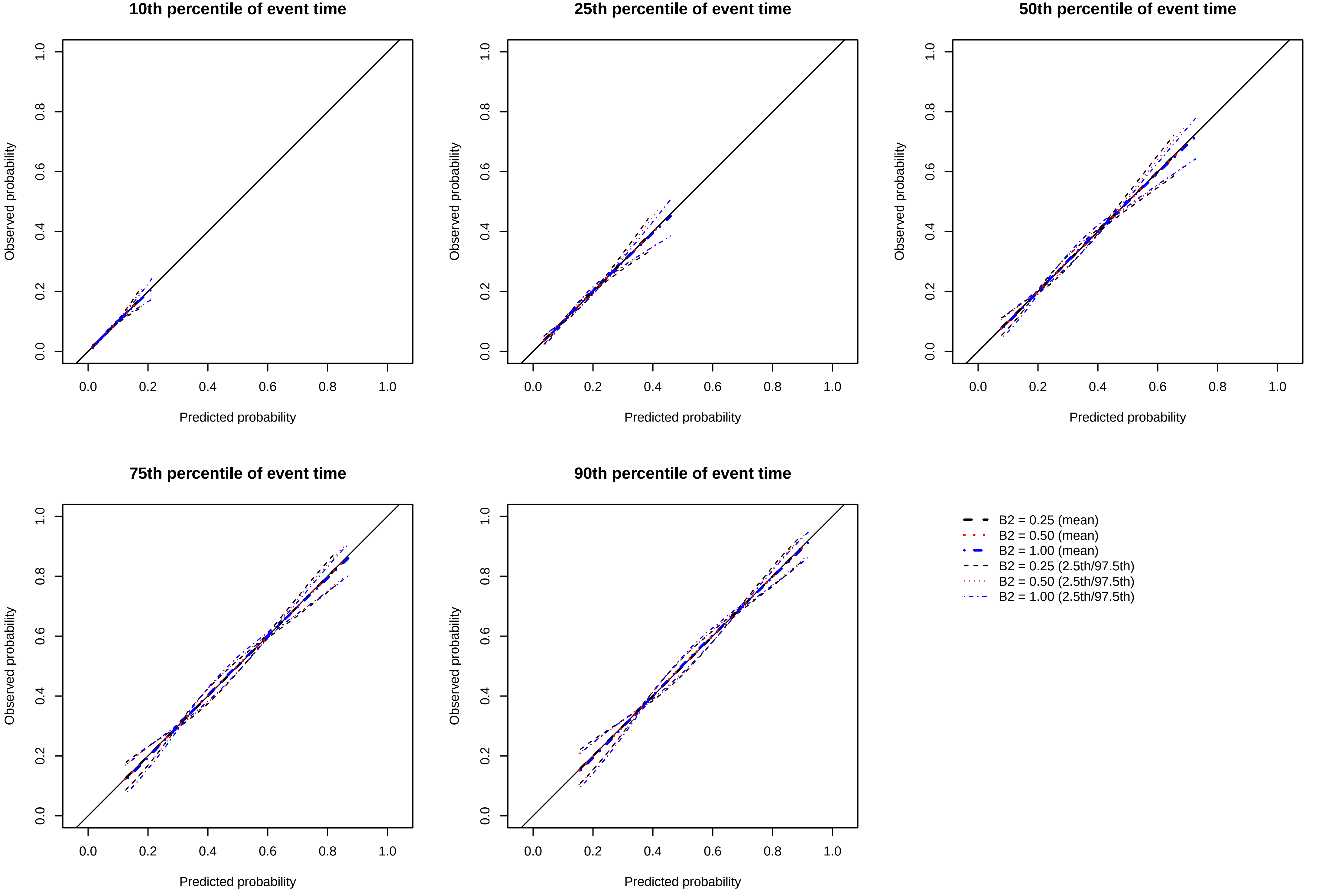

Figure A30. Mis−specified model (omission of main effect) (rho=0.50)

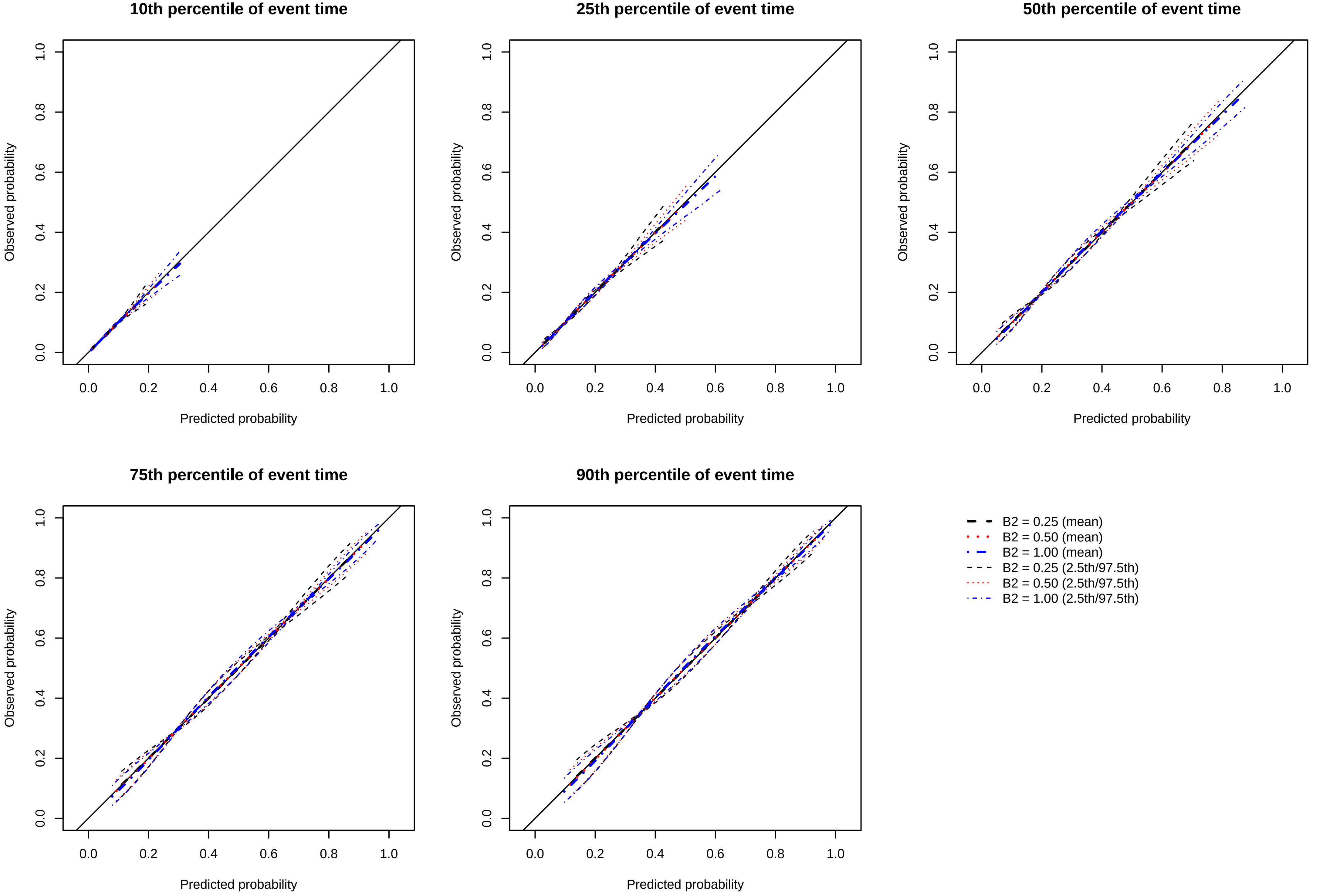

Figure A31. Mis−specified model (omission of main effect) (rho=0.75)

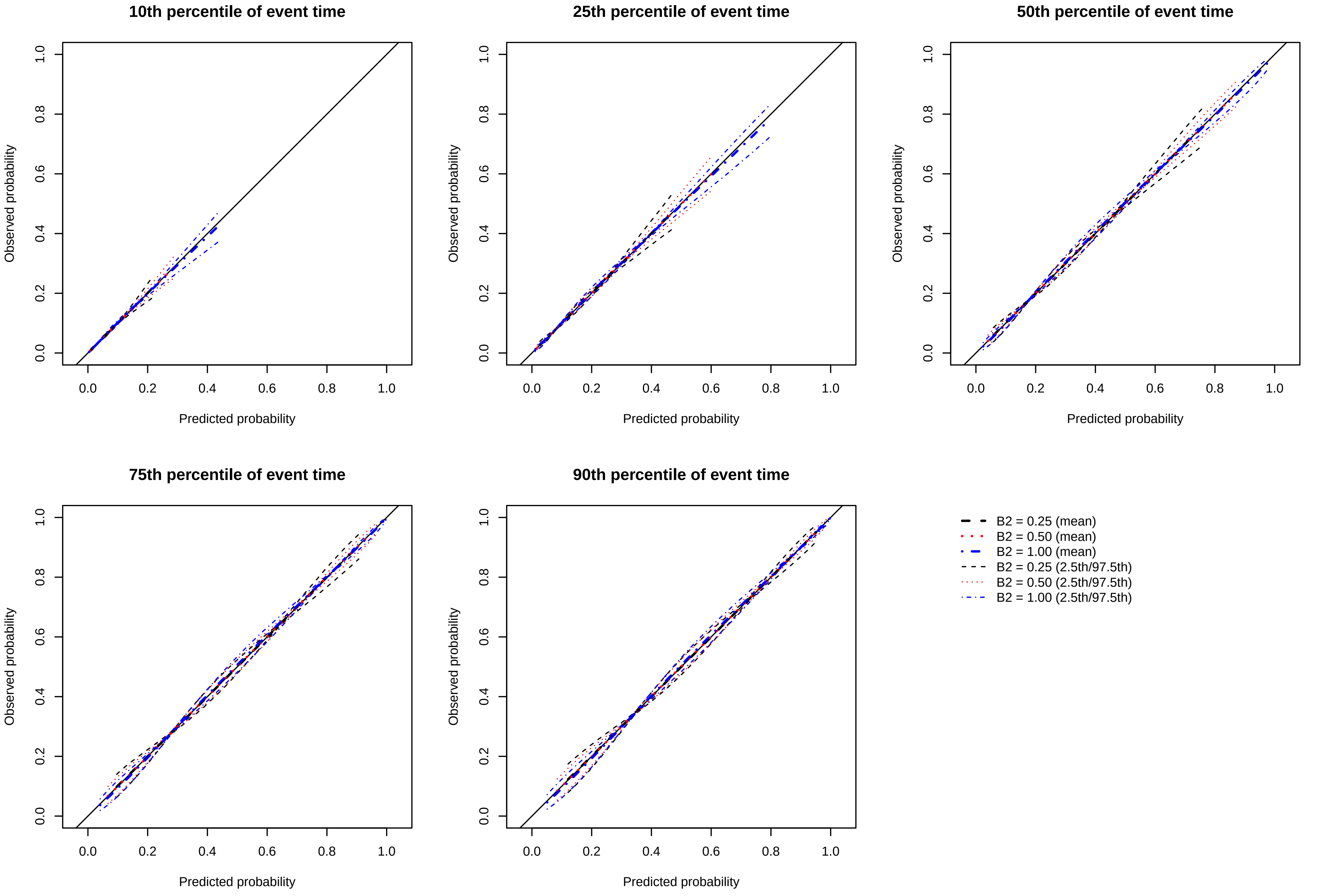

Supplement: Supplementary file 1 — Additional file 1. Figure S1. RCS: Choice of number of knots (p = 0.25). Figure S2. RCS: Choice of number of knots (p = 0.75). Figure S3. ICI/E50/E90 in simulations for selecting the optimal number of knots (p = 0.25). Figure S4. ICI/E50/E90 in simulations for selecting the optimal number of knots (p = 0.75). Figure S5. Effect of degree of censoring on estimated calibration curves (N = 2000 and p = 0.25). Figure S6. Effect of degree of censoring on estimated calibration curves (N = 2000 and p = 0.75). Figure S7. ICI/E90/E90 for correctly−specified model and censoring (N = 2000 and p = 0.25). Figure S8. ICI/E90/E90 for correctly−specified model and censoring (N = 2000 and p = 0.75). Figure S9. True model fitted with no censoring (beta1 = 0.25 & p = 0.25). Figure S10. True model fitted with no censoring (beta1 = 0.25 & p = 0.50). Figure S11. True model fitted with no censoring (beta1 = 0.25 & p = 0.75). Figure S12. True model fitted with no censoring (beta1 = 0.50 & p = 0.25). Figure S13. True model fitted with no censoring (beta1 = 0.50 & p = 0.75). Figure S14. True model fitted with no censoring (beta1 = 1 & p = 0.25). Figure S15. True model fitted with no censoring (beta1 = 1 & p = 0.50). Figure S16. True model fitted with no censoring (beta1 = 1 & p = 0.75). Figure S17. ICI/E90/E90 for correctly−specified model without censoring (p = 0.25). Figure S18. ICI/E90/E90 for correctly−specified model without censoring (p = 0.75). Figure S19. Mis−specified model (beta1 = 0.25 & p = 0.25). Figure S20. Mis−specified model (beta1 = 0.25 & p = 0.50). Figure S21. Mis−specified model (beta1 = 0.25 & p = 0.75). Figure S22. Mis−specified model (beta1 = 0.50 & p = 0.25). Figure S23. Mis−specified model (beta1 = 0.50 & p = 0.75). Figure S24. Mis−specified model (beta1 = 1 & p = 0.25). Figure S25. Mis−specified model (beta1 = 1 & p = 0.50). Figure S26. Mis−specified model (beta1 = 1 & p = 0.75). Figure S27. ICI/E90/E90 for incorrectly−specified model (p = 0.25). Figure S28. ICI/ [file 41512_2021_114_MOESM1_ESM.pdf]
